# Description of a species of *Fabaeformiscandona* (Ostracoda, Crustacea) from Kushiro Marsh, Hokkaido, Japan, with the nearly complete mitochondrial genomic sequence

**DOI:** 10.3897/BDJ.3.e7074

**Published:** 2015-12-11

**Authors:** Shimpei F. Hiruta, Shin-ichi Hiruta

**Affiliations:** ‡ Faculty of Science, Hokkaido University, Sapporo, Japan; § Department of Biology, Hokkaido University of Education, Kushiro Campus, Kushiro, Japan

**Keywords:** Kushiro Marsh, freshwater, Ostracoda, mitochondrial genome

## Abstract

**Background:**

So far, 16 species of non-marine ostracods have been reported from Kushiro Marsh, Kushiro Shitsugen National Park, eastern Hokkaido, Japan (Hiruta and Smith 2001, Smith and Hiruta 2004). Nine of these species are in Candonidae, the second-most diverse family of non-marine ostracods. This family contains ca. 550 species, or around 25% of the total number of non-marine ostracod species (Martens et al. 2008).

**New information:**

We sampled ostracods in Kushiro Marsh on 27 December 2012 and identified an undescribed species in the family Candonidae, herein described as *Fabaeformiscandona kushiroensis* sp. nov. This species belongs to the *F. acuminata* species group and is characterized by the shapes of the elongate, dorsally directed medial and outer lobes on the distal end of each hemipenis. We also determined for this species the sequence of the nearly complete mitochondrial genome, the first record from the order Podocopa. The genome (ca. 17 kbp) contains two ribosomal RNA, 22 transfer RNA, and 13 protein-coding genes, as also found in other arthropods for which the mitochondrial genome has been sequenced. The gene arrangement is similar to the pancrustacean ground pattern, except that in the control region there is an approximately 2 kbp tandem repeat region composed of 220-bp motif sequences. We describe the genetic features of the mitochondrial genome, including nucleotide composition and the secondary structures of tRNAs and rRNAs, and compare them with the genome of *Vargula hilgendorfii* (Myodocopa, Ostracoda).

## Introduction

Ostracods are small crustaceans, with most species being approximately one millimeter in length, which have a bivalved carapace covering non-mineralized body and appendages. They occur in almost every aquatic environment, including marine, brackish-water, freshwater, and groundwater. Ostracods have the most complete and continuous fossil record of any extant arthropod group, attributable to small body size, the calcified valves, and large population sizes (Moore 1961). The fossil record shows that the extant order-level lineages were already established by around 500 Ma, within ca. 50 million years (Maddocks 1982, Martens et al. 1998). Most traditional classifications in the Linnean system have ranked the Ostracoda at the class level (e.g., Martin and Davis 2001, Forest 2004), although recent phylogenomic studies (Regier et al. 2010, Oakley et al. 2013) have placed the group in the clade Oligostraca, together with Mystacocarida, Branchiura, and Pentastomida. In these molecular phylogenetic studies, Myodocopa and Podocopa were poorly resolved among arthropods and showed deep divergence, although these groups might be sister taxa, and there is no contradictory evidence for that.

**Figure 1.**
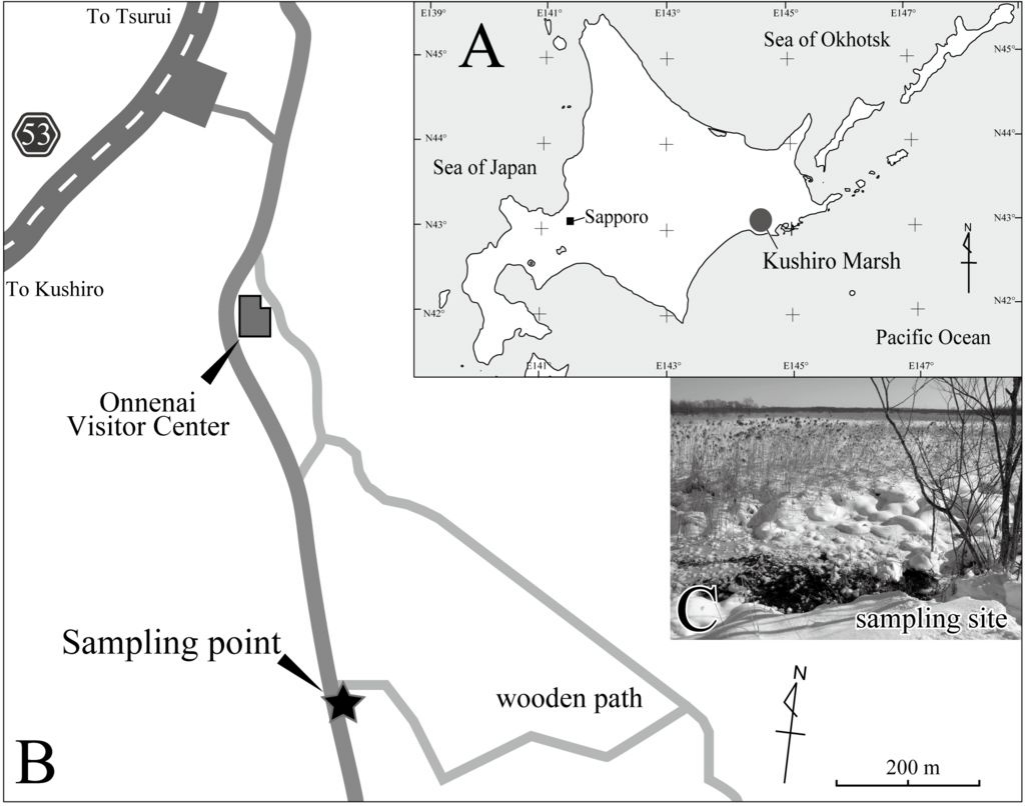
A, map of Hokkaido, Japan, showing the location of Kushiro Marsh; B, diagram of the study area near the Onnenai visitor center in Kushiro Marsh, with the sampling site indicated by star; C, photograph of the sampling site, small spring.

Kushiro Marsh, situated in Kushiro Shitsugen National Park, eastern Hokkaido, is the largest marshland in Japan (Fig. 1). It covers an area of 269 km^2^, which is 60% of the total area of Japan’s freshwater wetlands. Most of the shallow waters in the marsh are frozen from December to February. Previous studies have reported 16 species of non-marine ostracods from Kushiro Marsh (Hiruta and Smith 2001, Smith and Hiruta 2004), nine of which are in the family Candonidae, the second-most diverse family of non-marine ostracods. Candonidae contains around 550 species, or roughly 25% of the total number of non-marine ostracod species (Martens et al. 2008).

Although complete mitochondrial genomic sequences are useful for phylogenetic and population genetic studies (e.g., Kilpert et al. 2012, Omote et al. 2013), only one has been determined from ostracods. Ogoh and Ohmiya (2004) reported the complete mitochondrial genomic sequence for the sea-firefly, *Vargula hilgendorfii* (Müller 1890) (Myodocopa, Ostracoda), but no complete mitochondrial genomic sequence was available for any species in Podocopa. In this report, we describe a new species in *Fabaeformiscandona* and report its nearly complete mitochondrial genomic sequence, the first record from the order Podocopa. We also describe the features of the mitochondrial genome and compare them with the genome of *V. hilgendorfii*.

## Materials and methods

*Specimens*

Material was collectednear the Onnenai Visitor Center (43°06′17.6″N 144°19′46.5″E) in Kushiro Marsh, Hokkaido, Japan (Fig. 1) on 27 December 2012, when the ambient temperature was −10°C. The sampling site is located in a spring area beside a hill. Water and bottom sediment were strained through a 0.1-mm-mesh sieve. Specimens were sorted under a stereoscopic microscope and preserved in 99% ethanol.

Selected specimens were dissected, and the appendages were mounted in Hoyer’s solution on glass slides and drawn with the aid of a camera lucida. Some carapaces were pasted with a tragacanth gum solution onto microfossil slides. For scanning electron microscope (SEM) observation, carapaces and soft parts were mounted on stub after treatment with hexamethyldisilazane (HMDS) (Nation 1983). Specimens were coated with gold and examined with an S-3000N (Hitachi High Technologies) SEM at 15–20 kV accelerating voltage. The material used in this study has been deposited in the Invertebrate Collection of the Hokkaido University Museum, Sapporo (ICHUM).

The chaetotaxic notation follows that of Broodbakker and Danielopol (1982), as revised for the antennae by Martens (1987) and for the thoracopods by Meisch (1996). Hemipenis terminology follows that of Danielopol (1969). We use the same abbreviations for limbs as Meisch (2000) and Smith and Janz (2008).

*List of abbreviations*

*a*, outer lobe of hemipenis; A1, antennule; A2, antenna; *b*, inner lobe of hemipenis; *E*, endopod; *Exo*, exopod; FRO, female reproductive organ; *G1*–*3*, apical claws on penultimate segment of antenna; *GM*, *GM*, apical claws on terminal segment of antenna; *h*, medial lobe of hemipenis; Hp, hemipenis; L5, maxilliped; L6, walking leg; L7, cleaning leg; LV, left valve; Md, mandible; M-process, chitinized internal process of hemipenis; Mx, maxillula; *Pr*, protopod; RV, right valve; *Y*, *y2*, *y3*, aesthetascs on antenna; *ya*, aesthetasc of antennule.

*DNA extraction*

Total genomic DNA was extracted from the whole body of one individual by using a DNeasy Blood & Tissue Kit (QIAGEN), with modifications from Johnson et al. (2004). Specimens were incubated in ATL buffer with proteinase K for at least 48 h at 55°C to lyse the tissue. Before the lysis mixture was pipetted into a spin column, the exoskeleton and carapaces of the specimen were retrieved and mounted in Hoyer’s solution on a glass slide.

*Amplification of partial mitochondrial gene sequences*

Initially, universal primer sets were used to amplify parts of the cytochrome *c* oxidase subunit I gene (*COI*) and 12S rRNA (srRNA) genes (primers LCO1490 and HCO2198 for *COI*, Folmer et al. 1994; 121Sa and 12Sb for srRNA, Palumbi 1996). PCRs were performed in an ABI 2720 Thermal Cycler (Applied Biosystems) in 10-µl volumes containing 1 µl of template solution, 0.8 µl of 2.5 mM each dNTP, 10 pmol of each primer, and 0.25 U Ex *Taq* polymerase (Takara) in 1× buffer (Takara). Amplification conditions for *COI* and srRNA were 95°C for 1 min; 35 cycles of 95°C for 30 sec, 50°C for 30 sec, and 72°C for 1 min; and 72°C for 7 min.

*Amplification of the whole mitochondrial genome*

The *COI* and srRNA sequences were used to design new primer sets (Suppl. material 5; Fab_MtF_COIF, Fab_MtF_COIR, Fab_MtF_12S, and Fab_MtF_12R), which were used for long-PCR amplification of the whole mitochondrial genome in two parts. Long-PCRs were carried out in 50-µl reaction volumes containing 1 µl of template solution, 4 µl of 2.5 mM each dNTP, 10 pmol of each primer, and 1.25 U PrimeSTAR GXL DNA polymerase (Takara) in 1× buffer (Takara). Amplification conditions for the two fragments were 30 cycles of 98°C for 10 sec and 68°C for 10 min; and 68°C for 7 min. Primers used for primer walking to sequence the long amplicons obtained are listed in Suppl. material 5. The direction and position of each primer is shown in Suppl. material 1.

*Amplification of nuclear rRNA genes*

Nuclear rRNA genes were amplified with primer set 18S_F1 and Mallat_R. Long-PCR in 50-µl volumes containing 1 µl of template solution, 4 µl of 2.5 mM each dNTP, 10 pmol of each primer, and 1.25 U PrimeSTAR GXL DNA polymerase (Takara) in 1× buffer (Takara). Amplification conditions were as for the whole mitochondrial genome fragments. Internal primers used for sequencing the nuclear fragments are listed in Suppl. material 5.

*Sequencing*

Amplification products were purified by the method of Boom et al. (1990). Nucleotide sequences were determined by direct sequencing using a BigDye Terminator Cycle Sequencing Kit ver. 3.1 and an ABI 3730 DNA analyzer (Applied Biosystems). Sequences have been deposited in Genbank under accession numbers AP014656 (mitochondrial DNA) and AB996740 (nuclear rRNA).

*Length estimation for the tandem-repeat region*

To estimate the length of tandem-repeat region within the control region (CR), primers were designed (Fab_CRF and Fab_CRmF; Suppl. material 5) that bound close to the repeat region on either side. The repeat region was amplified by PCR and its length estimated by electrophoresis.

*Sequence analysis*

Nucleotide sequences were assembled and edited with MEGA5 (Tamura et al. 2011). Each gene in the mitochondrial genome was identified on the MITOS Web Server (Bernt et al. 2013). Area of *ND4* and *COIII* genes were detected into separated regions by using MITOS Web server, so that we conformed the nucleotides and the translated amino acid sequences to determine each of the coding area. The putative secondary structures of the tRNA genes were also estimated by MITOS Web server.

The boundaries and secondary structures of the mt rRNA genes were determined by using Centroidfold (Sato et al. 2009).

For the nuclear rRNA genes, information on secondary structure from *Apis mellifera* (Gillespie et al. 2006) and the secondary structure estimated by Centroidfold were used to determine the boundaries of each gene.

## Taxon treatments

### Fabaeformiscandona kushiroensis

Hiruta & Hiruta sp. n.

AP014656

AB996740

urn:lsid:zoobank.org:act:8C3A16C1-50EC-4A9C-B480-56E89FCC30C9

#### Materials

- **Type status:** Holotype. **Occurrence:** catalogNumber: ICHUM_5034; recordedBy: Shimpei F. Hiruta; individualCount: 1; sex: male; lifeStage: adult; preparations: soft parts mounted on 10 slides, carapace mounted on a microfossil slide; **Taxon:** scientificName: Fabaeformiscandona kushiroensis; **Location:** country: Japan; stateProvince: Hokkaido; locality: Kushiro Marsh National Park, Hokkaido, Japan; verbatimElevation: 10 m; verbatimCoordinates: 43°6′17.6″N, 144°19′46.5″E; decimalLatitude: 43.105; decimalLongitude: 144.3291; georeferenceProtocol: GPS; **Identification:** identifiedBy: Shimpei F. Hiruta; dateIdentified: 2013; **Event:** samplingProtocol: Strained by 0.1 mm mesh sieve; eventDate: 12/27/2012; **Record Level:** language: en; collectionID: ICHUM 5034; institutionCode: Invertebrate Collection of the Hokkaido University Museum (ICHUM); collectionCode: Ostracoda; basisOfRecord: PreservedSpecimen
- **Type status:** Paratype. **Occurrence:** catalogNumber: ICHUM_5035; recordedBy: Shimpei F. Hiruta; individualCount: 1; sex: female; lifeStage: adult; preparations: soft parts mounted on 8 slides, carapaces mounted on a microfossil slide.; **Taxon:** scientificName: Fabaeformiscandona kushiroensis; **Location:** country: Japan; stateProvince: Hokkaido; locality: Kushiro Marsh National Park, Hokkaido, Japan; verbatimElevation: 10 m; verbatimCoordinates: 43°6′17.6″N, 144°19′46.5″E; decimalLatitude: 43.105; decimalLongitude: 144.3291; georeferenceProtocol: GPS; **Identification:** identifiedBy: Shimpei F. Hiruta; dateIdentified: 2013; **Event:** samplingProtocol: Strained by 0.1 mm mesh sieve; eventDate: 12/27/2012; **Record Level:** language: en; collectionID: ICHUM 5035; institutionCode: Invertebrate Collection of the Hokkaido University Museum (ICHUM); collectionCode: Ostracoda; basisOfRecord: PreservedSpecimen
- **Type status:** Paratype. **Occurrence:** catalogNumber: ICHUM_5036; recordedBy: Shimpei F. Hiruta; individualCount: 1; sex: male; lifeStage: adult; preparations: mounted on a slide; **Taxon:** scientificName: Fabaeformiscandona kushiroensis; **Location:** country: Japan; stateProvince: Hokkaido; locality: Kushiro Marsh National Park, Hokkaido, Japan; verbatimElevation: 10 m; verbatimCoordinates: 43°6′17.6″N, 144°19′46.5″E; decimalLatitude: 43.105; decimalLongitude: 144.3291; georeferenceProtocol: GPS; **Identification:** identifiedBy: Shimpei F. Hiruta; dateIdentified: 2013; **Event:** samplingProtocol: Strained by 0.1 mm mesh sieve; eventDate: 12/27/2012; **Record Level:** language: en; collectionID: ICHUM 5036; institutionCode: Invertebrate Collection of the Hokkaido University Museum (ICHUM); collectionCode: Ostracoda; basisOfRecord: PreservedSpecimen
- **Type status:** Paratype. **Occurrence:** catalogNumber: ICHUM_5037; recordedBy: Shimpei F. Hiruta; individualCount: 1; sex: male; lifeStage: adult; preparations: mounted on a slide; **Taxon:** scientificName: Fabaeformiscandona kushiroensis; **Location:** country: Japan; stateProvince: Hokkaido; locality: Kushiro Marsh National Park, Hokkaido, Japan; verbatimElevation: 10 m; verbatimCoordinates: 43°6′17.6″N, 144°19′46.5″E; decimalLatitude: 43.105; decimalLongitude: 144.3291; georeferenceProtocol: GPS; **Identification:** identifiedBy: Shimpei F. Hiruta; dateIdentified: 2013; **Event:** samplingProtocol: Strained by 0.1 mm mesh sieve; eventDate: 12/27/2012; **Record Level:** language: en; collectionID: ICHUM 5037; institutionCode: Invertebrate Collection of the Hokkaido University Museum (ICHUM); collectionCode: Ostracoda; basisOfRecord: PreservedSpecimen
- **Type status:** Paratype. **Occurrence:** catalogNumber: ICHUM_5038; recordedBy: Shimpei F. Hiruta; individualCount: 1; sex: female; lifeStage: adult; preparations: mounted on a slide; **Taxon:** scientificName: Fabaeformiscandona kushiroensis; **Location:** country: Japan; stateProvince: Hokkaido; locality: Kushiro Marsh National Park, Hokkaido, Japan; verbatimElevation: 10 m; verbatimCoordinates: 43°6′17.6″N, 144°19′46.5″E; decimalLatitude: 43.105; decimalLongitude: 144.3291; georeferenceProtocol: GPS; **Identification:** identifiedBy: Shimpei F. Hiruta; dateIdentified: 2013; **Event:** samplingProtocol: Strained by 0.1 mm mesh sieve; eventDate: 12/27/2012; **Record Level:** language: en; collectionID: ICHUM 5038; institutionCode: Invertebrate Collection of the Hokkaido University Museum (ICHUM); collectionCode: Ostracoda; basisOfRecord: PreservedSpecimen
- **Type status:** Paratype. **Occurrence:** catalogNumber: ICHUM_5039; recordedBy: Shimpei F. Hiruta; individualCount: 1; sex: male; lifeStage: adult; preparations: mounted on a stub for SEM observation; **Taxon:** scientificName: Fabaeformiscandona kushiroensis; **Location:** country: Japan; stateProvince: Hokkaido; locality: Kushiro Marsh National Park, Hokkaido, Japan; verbatimElevation: 10 m; verbatimCoordinates: 43°6′17.6″N, 144°19′46.5″E; decimalLatitude: 43.105; decimalLongitude: 144.3291; georeferenceProtocol: GPS; **Identification:** identifiedBy: Shimpei F. Hiruta; dateIdentified: 2013; **Event:** samplingProtocol: Strained by 0.1 mm mesh sieve; eventDate: 12/27/2012; **Record Level:** language: en; collectionID: ICHUM 5039; institutionCode: Invertebrate Collection of the Hokkaido University Museum (ICHUM); collectionCode: Ostracoda; basisOfRecord: PreservedSpecimen
- **Type status:** Paratype. **Occurrence:** catalogNumber: ICHUM_5040; recordedBy: Shimpei F. Hiruta; individualCount: 1; sex: female; lifeStage: adult; preparations: mounted on a stub for SEM observation; **Taxon:** scientificName: Fabaeformiscandona kushiroensis; **Location:** country: Japan; stateProvince: Hokkaido; locality: Kushiro Marsh National Park, Hokkaido, Japan; verbatimElevation: 10 m; verbatimCoordinates: 43°6′17.6″N, 144°19′46.5″E; decimalLatitude: 43.105; decimalLongitude: 144.3291; georeferenceProtocol: GPS; **Identification:** identifiedBy: Shimpei F. Hiruta; dateIdentified: 2013; **Event:** samplingProtocol: Strained by 0.1 mm mesh sieve; eventDate: 12/27/2012; **Record Level:** language: en; collectionID: ICHUM 5040; institutionCode: Invertebrate Collection of the Hokkaido University Museum (ICHUM); collectionCode: Ostracoda; basisOfRecord: PreservedSpecimen
- **Type status:** Paratype. **Occurrence:** catalogNumber: ICHUM_5041; recordedBy: Shimpei F. Hiruta; individualCount: 1; sex: female; lifeStage: adult; preparations: mounted on a stub for SEM observation; **Taxon:** scientificName: Fabaeformiscandona kushiroensis; **Location:** country: Japan; stateProvince: Hokkaido; locality: Kushiro Marsh National Park, Hokkaido, Japan; verbatimElevation: 10 m; verbatimCoordinates: 43°6′17.6″N, 144°19′46.5″E; decimalLatitude: 43.105; decimalLongitude: 144.3291; georeferenceProtocol: GPS; **Identification:** identifiedBy: Shimpei F. Hiruta; dateIdentified: 2013; **Event:** samplingProtocol: Strained by 0.1 mm mesh sieve; eventDate: 12/27/2012; **Record Level:** language: en; collectionID: ICHUM 5041; institutionCode: Invertebrate Collection of the Hokkaido University Museum (ICHUM); collectionCode: Ostracoda; basisOfRecord: PreservedSpecimen
- **Type status:** Paratype. **Occurrence:** catalogNumber: ICHUM_5042; recordedBy: Shimpei F. Hiruta; individualCount: 1; sex: female; lifeStage: adult; preparations: mounted on a stub for SEM observation; **Taxon:** scientificName: Fabaeformiscandona kushiroensis; **Location:** country: Japan; stateProvince: Hokkaido; locality: Kushiro Marsh National Park, Hokkaido, Japan; verbatimElevation: 10 m; verbatimCoordinates: 43°6′17.6″N, 144°19′46.5″E; decimalLatitude: 43.105; decimalLongitude: 144.3291; georeferenceProtocol: GPS; **Identification:** identifiedBy: Shimpei F. Hiruta; dateIdentified: 2013; **Event:** samplingProtocol: Strained by 0.1 mm mesh sieve; eventDate: 12/27/2012; **Record Level:** language: en; collectionID: ICHUM 5042; institutionCode: Invertebrate Collection of the Hokkaido University Museum (ICHUM); collectionCode: Ostracoda; basisOfRecord: PreservedSpecimen
- **Type status:** Paratype. **Occurrence:** catalogNumber: ICHUM_5043; recordedBy: Shimpei F. Hiruta; individualCount: 1; sex: male; lifeStage: adult; preparations: exoskeleton mounted on a slide after DNA extraction; associatedSequences: GenBank: AP014656, AB996740; **Taxon:** scientificName: Fabaeformiscandona kushiroensis; **Location:** country: Japan; stateProvince: Hokkaido; locality: Kushiro Marsh National Park, Hokkaido, Japan; verbatimElevation: 10 m; verbatimCoordinates: 43°6′17.6″N, 144°19′46.5″E; decimalLatitude: 43.105; decimalLongitude: 144.3291; georeferenceProtocol: GPS; **Identification:** identifiedBy: Shimpei F. Hiruta; dateIdentified: 2013; **Event:** samplingProtocol: Strained by 0.1 mm mesh sieve; eventDate: 12/27/2012; **Record Level:** language: en; collectionID: ICHUM 5043; institutionCode: Invertebrate Collection of the Hokkaido University Museum (ICHUM); collectionCode: Ostracoda; basisOfRecord: PreservedSpecimen
- **Type status:** Paratype. **Occurrence:** catalogNumber: ICHUM_5044; recordedBy: Shimpei F. Hiruta; individualCount: 50; sex: undetermined; lifeStage: adult; preparations: exoskeleton preserved in 99% ethanol after DNA extraction; **Taxon:** scientificName: Fabaeformiscandona kushiroensis; **Location:** country: Japan; stateProvince: Hokkaido; locality: Kushiro Marsh National Park, Hokkaido, Japan; verbatimElevation: 10 m; verbatimCoordinates: 43°6′17.6″N, 144°19′46.5″E; decimalLatitude: 43.105; decimalLongitude: 144.3291; georeferenceProtocol: GPS; **Identification:** identifiedBy: Shimpei F. Hiruta; dateIdentified: 2013; **Event:** samplingProtocol: Strained by 0.1 mm mesh sieve; eventDate: 12/27/2012; **Record Level:** language: en; collectionID: ICHUM 5044; institutionCode: Invertebrate Collection of the Hokkaido University Museum (ICHUM); collectionCode: Ostracoda; basisOfRecord: PreservedSpecimen
- **Type status:** Paratype. **Occurrence:** catalogNumber: ICHUM_5116; recordedBy: Shimpei F. Hiruta; individualCount: 3; sex: 1 male, 2 females; lifeStage: adult; preparations: mounted on a stub for SEM observation; **Taxon:** scientificName: Fabaeformiscandona kushiroensis; **Location:** country: Japan; stateProvince: Hokkaido; locality: Kushiro Marsh National Park, Hokkaido, Japan; verbatimElevation: 10 m; verbatimCoordinates: 43°6′17.6″N, 144°19′46.5″E; decimalLatitude: 43.105; decimalLongitude: 144.3291; georeferenceProtocol: GPS; **Identification:** identifiedBy: Shimpei F. Hiruta; dateIdentified: 2013; **Event:** samplingProtocol: Strained by 0.1 mm mesh sieve; eventDate: 12/27/2012; **Record Level:** language: en; collectionID: ICHUM 5116; institutionCode: Invertebrate Collection of the Hokkaido University Museum (ICHUM); collectionCode: Ostracoda; basisOfRecord: PreservedSpecimen

#### Description

*Description of male*.

**Figure 2.**
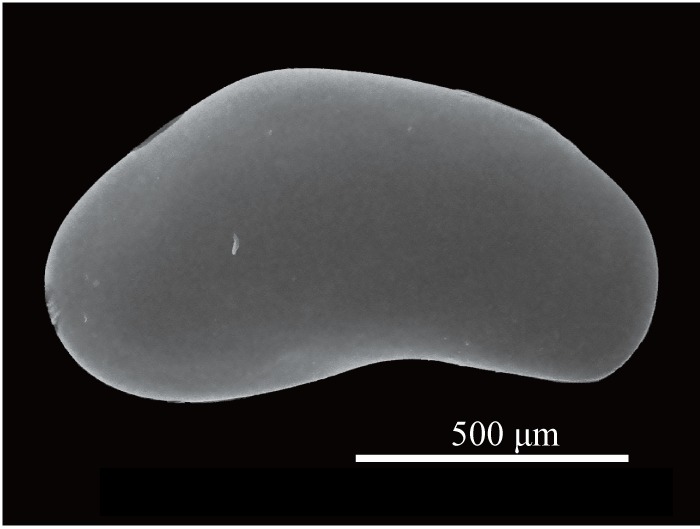
*Fabaeformiscandona kushiroensis* sp. n. SEM images. Paratype, ICHUM 5042, lateral view of male RV.

**Figure 3.**
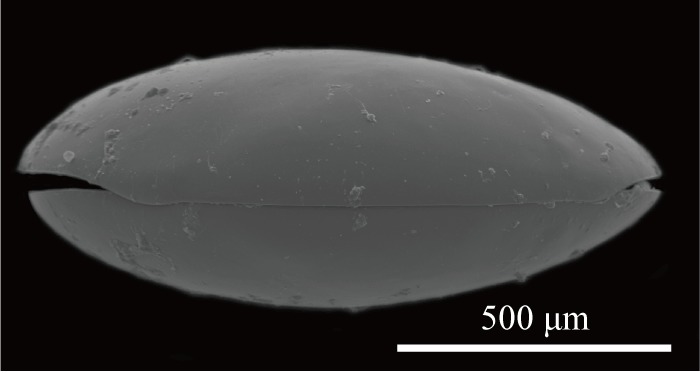
*Fabaeformiscandona kushiroensis* sp. n. SEM images. Paratype, ICHUM 5116, dorsal view of male carapace.

Carapace (Figs 2, 3, 7) 1.16–1.24 mm long, 0.62–0.66 mm high (n = 4; holotype 1.24 mm long, 0.66 mm high); greatest height situated at dorsal hump approximately one-third of length from posterior margin; either side of the dorsal humps almost straight; anterior and posterior margins equally rounded; ventral margin concave at two-fifths of length from anterior end. Calcified inner lamella wider anteriorly than posteriorly. Carapace laterally compressed in dorsal view; greatest width situated about at mid-length; LV overlapping to RV; postero-dorsal flap of LV broad and somewhat longer than antero-dorsal flap (Fig. 3; Suppl. material 4). Surface of valves smooth, color translucent white (Fig. 2).

A1 (Fig. 9) seven articulated segmented. First two podomeres fused, with two dorsal setae and two long, stout apico-ventral setae. Third podomere rectangular, with apico-dorsal seta. Fourth podomere quadrate, with one apico-dorsal seta. Fifth podomere quadrate, with one long apico-dorsal and one apico-ventral setae. Sixth podomere rectangular, with two long apico-dorsal and one apico-ventral setae. Seventh podomere elongate, with one long apico-dorsal and two apico-ventral setae. Eighth podomere elongate, slender, with two long and one shorter setae, and aesthetasc *ya.*

A2 (Fig. 10) five-segmented. First podomere (*Pr*) with one apico-ventral seta of medium length; one long antero-proximal plumose seta; one long antero-proximal seta; and one long latero-proximal seta. Second podomere (*EI*) with one apico-ventral seta, aesthetasc *Y*, and exopodite (*Exo*); exopodite consists of one long distally plumed and two short setae. Third podomere (*EII*) with two mid-apical male bristles (*t2* and *t3*), one short apico-ventral (*t1*) and one apico-dorsal (*t4*) setae, and aesthetasc *y2*. Fourth podomere (*EIII*) with *G1*, short *G2*, *z1*, and *z2* claws, and *G3* and *z3* setae. Fifth podomere (*EIV*) with *GM* and shorter *GM* claws, one apical seta, and aesthetasc *y3*.

Md (Fig. 11) consists of coxal plate and four-segmented palp. Coxal plate with antero-lateral seta and seven stout teeth, latter interspersed with several setae of various lengths. First podomere of palp with exopodal plate (*Exo*) and one long and one short, stout inner-distal plumose setae, one long antero-distal seta, and alpha simple seta. Second podomere of palp with beta seta, group of four setae, and one plumose seta. Third podomere of palp with three outer-apical setae, one mid-apical gamma seta, and two long apical setae. Fourth podomere of palp with two claws and two apical setae.

Mx (Figs 12, 13) with elongate vibratory plate, three masticatory processes, and two-segmented palp. Masticatory process with numerous setae. First podomere with three apical plumose setae.

L5 (Figs 14, 15) with palp, two filament-like setae (*Exo*), one antero-proximal seta, one antero-apical seta, and one postero-apical seta. Palp transformed into asymmetrical clasping processes; left clasping process (Fig. 14) slender, somewhat curved and hook-like in shape, with two short mid-setae; right clasping process (Fig. 15) sharply curved and proximally stout, helmet-like in shape, with two short mid-setae. Masticatory process with numerous setae.

L6 (Fig. 16) five-segmented. Apical setae (*e*, *f*, *g*) on second to fourth podomeres long, each extending past end of subsequent podomere. Terminal claw (*h2*) slender, long.

L7 (Fig. 17) five-segmented. Penultimate segment subdivided. First podomere with two setae (*d1*, *dp*). Fourth podomere with one apical seta (*g*). Fifth podomere with two long (*h2*, *h3*) and one shorter (*h1*) setae. One third of distal tips of the *h3* seta bend.

Uropodal ramus (Fig. 18) with short anterior seta (*Sa*) and well-developed posterior seta (*Sp*) longer than terminal claws. Two terminal claws simple, slender.

Hp (Fig. 19) distally with three lobes distally; median lobe (*h*) elongate, directed toward dorsal side, truncate distally; outer lobe (*a*) elongate, directed dorsally acute distally. Inner lobe (*b*) largely rounded and overlapping to the other two lobes. M-process well developed, S-shaped, rounded distally. Bursa copulatrix well-developed, pointed distally, with proximal bump.

Zenker’s organ (Fig. 20) with 5 + 2 internal rings of spines. Seminal vesicle located at anterior end.

*Description of female*.

**Figure 4.**
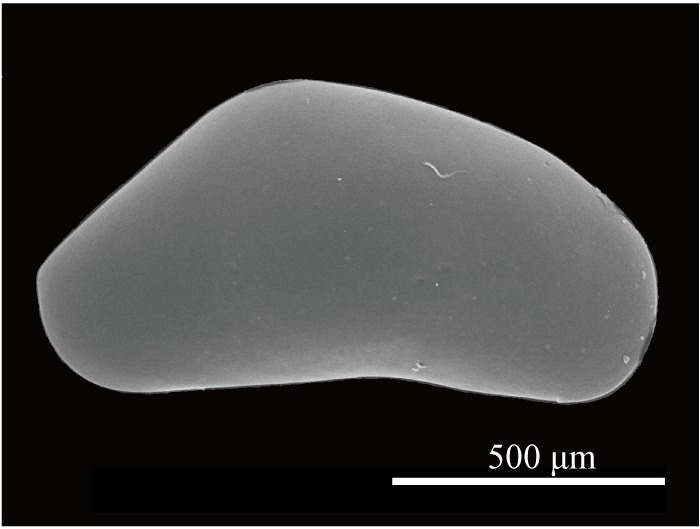
*Fabaeformiscandona kushiroensis* sp. n. SEM images. Paratype, ICHUM 5040, lateral view of female RV.

**Figure 5.**
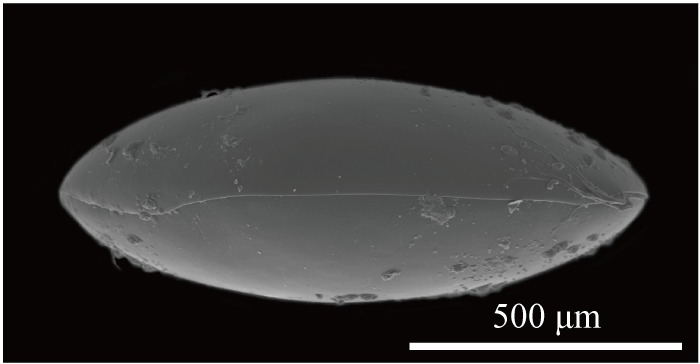
*Fabaeformiscandona kushiroensis* sp. n. SEM images. Paratype, ICHUM 5116, Dorsal view of female carapace.

**Figure 6.**
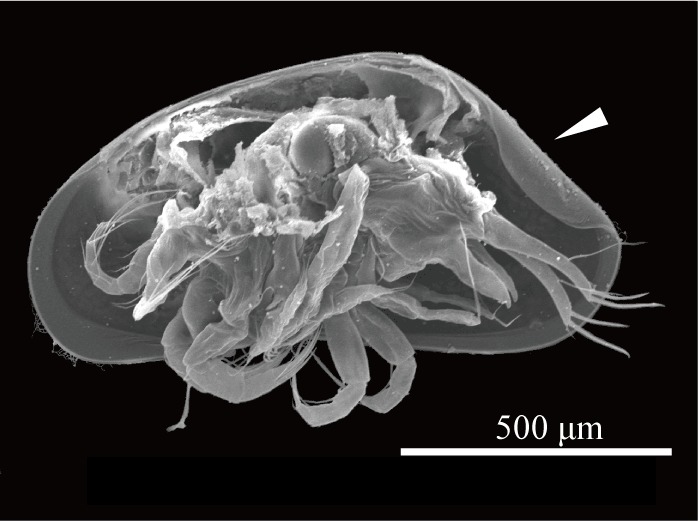
*Fabaeformiscandona kushiroensis* sp. n. SEM images. Paratype, ICHUM 5039, lateral view of female with LV removed. Arrow head shows postero-dorsal inner fold of female RV.

**Figure 7.**
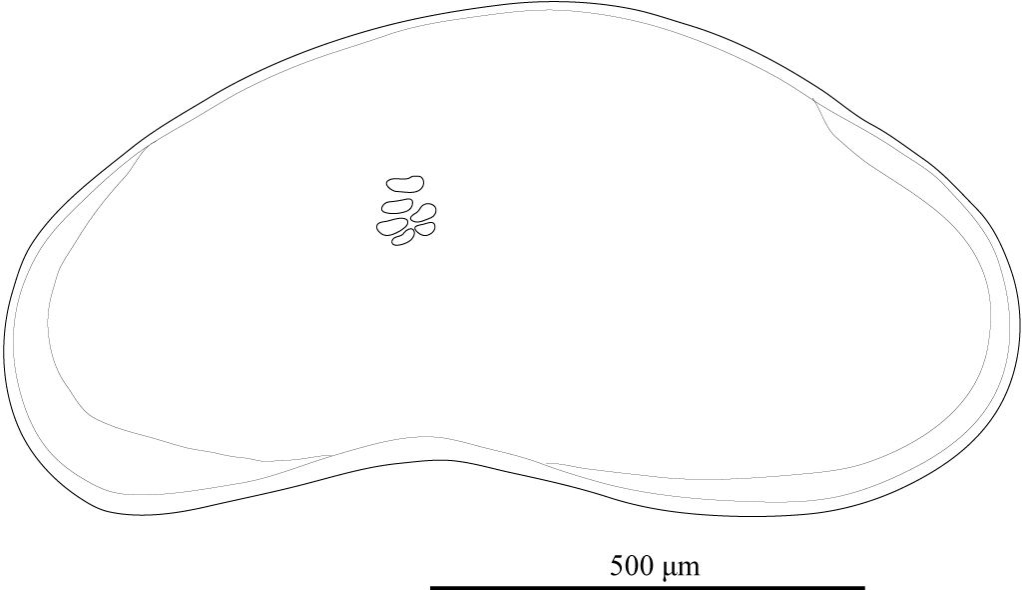
*Fabaeformiscandona kushiroensis* sp. n. Holotype, male, ICHUM 5034, Lateral view of RV.

**Figure 8.**
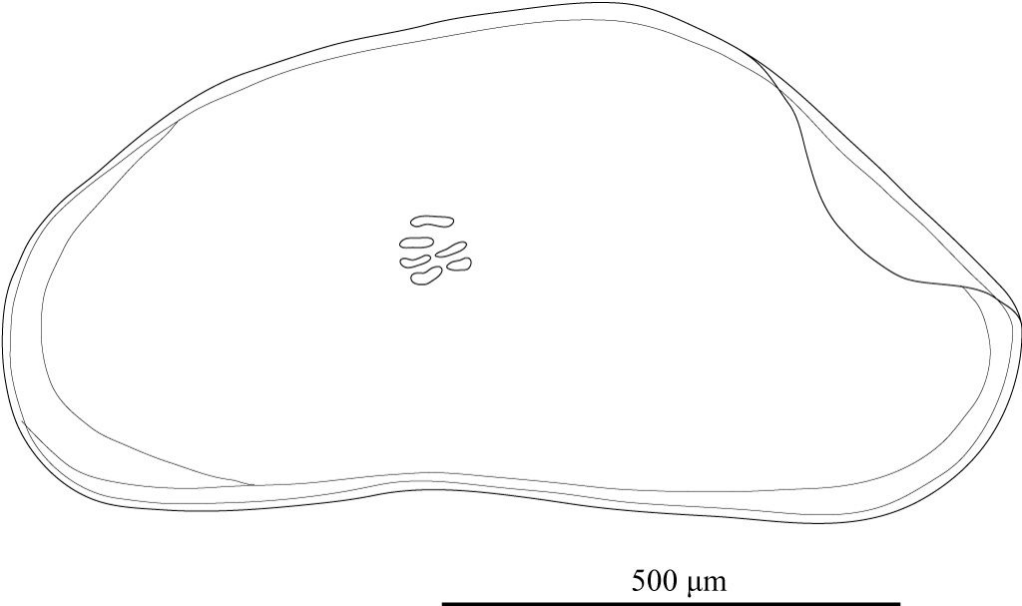
*Fabaeformiscandona kushiroensis* sp. n. Paratype, female, ICHUM 5035, Lateral view of RV.

**Figure 9.**
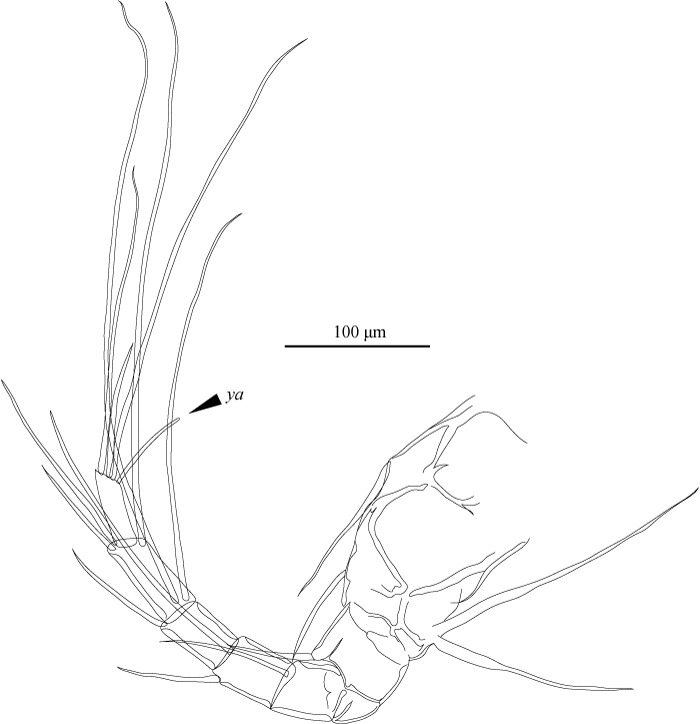
*Fabaeformiscandona kushiroensis* sp. n. Holotype, male, ICHUM 5034, A1.

**Figure 10.**
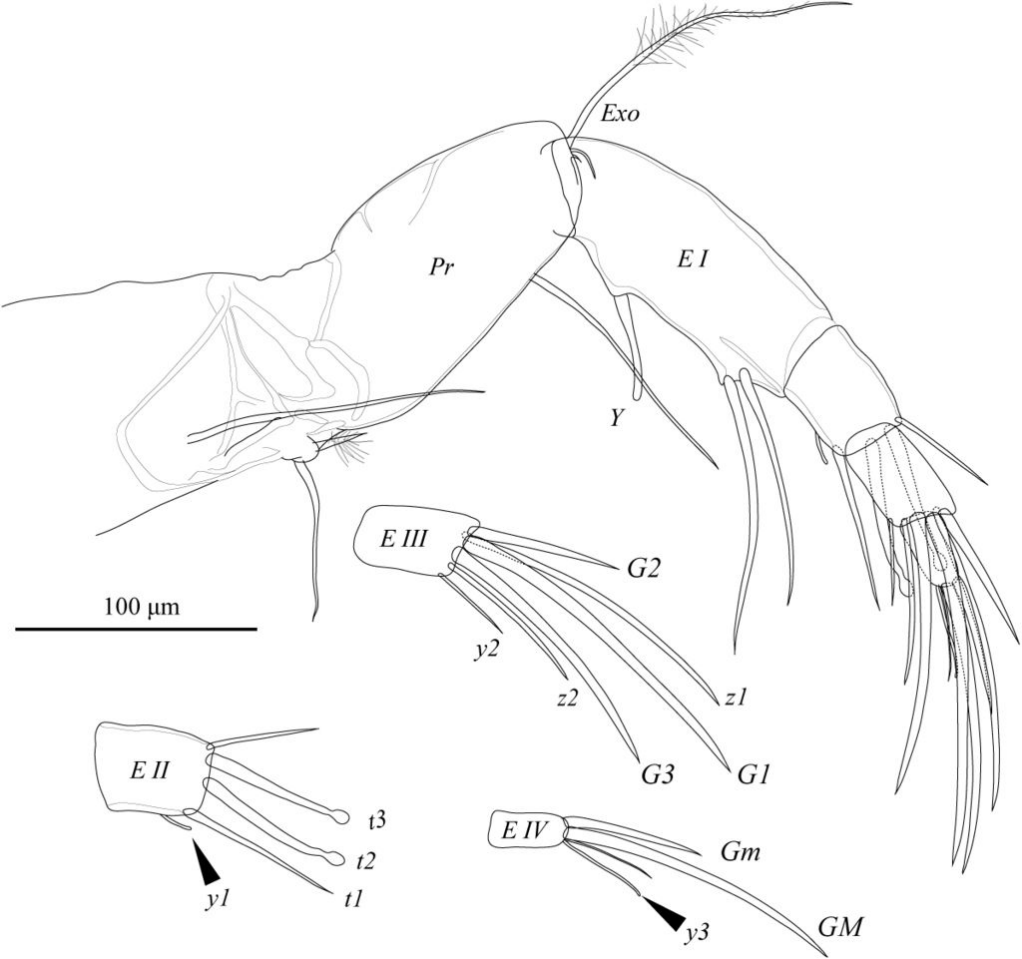
*Fabaeformiscandona kushiroensis* sp. n. Holotype, male, ICHUM 5034 Male A2 (inset, details of third to fifth podomeres).

**Figure 11.**
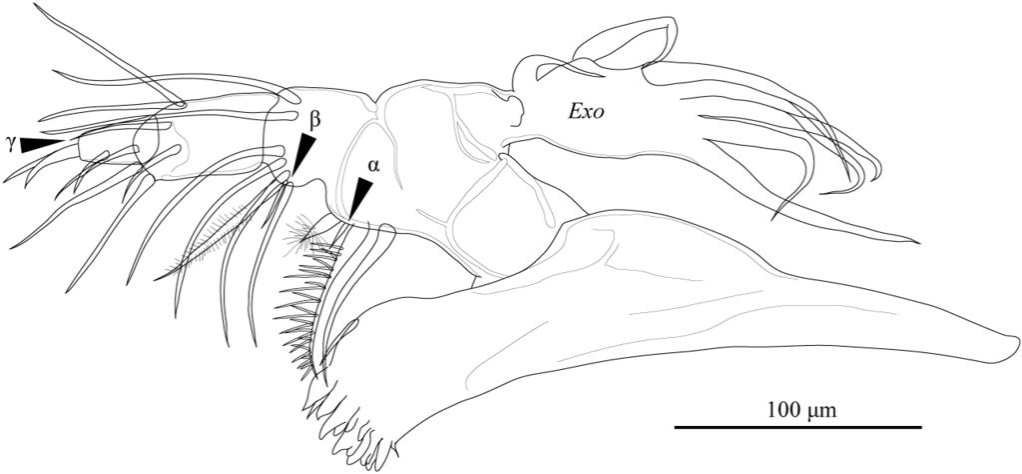
*Fabaeformiscandona kushiroensis* sp. n. Holotype, male, ICHUM 5034, Md.

**Figure 12.**
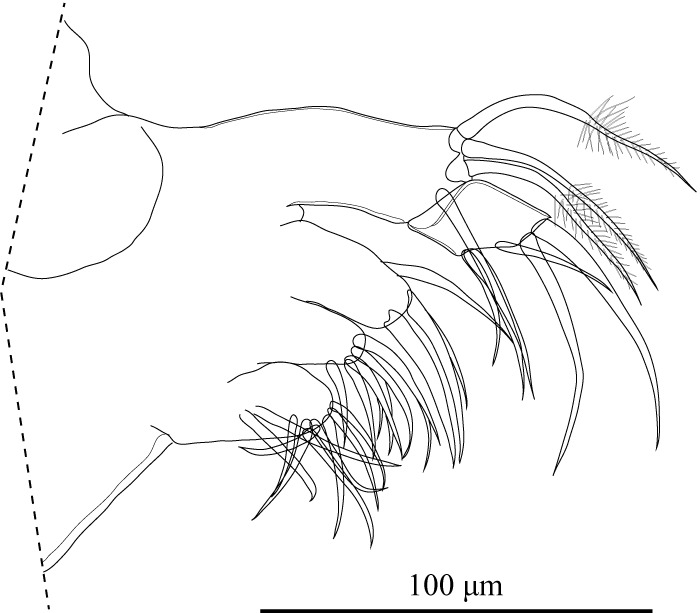
*Fabaeformiscandona kushiroensis* sp. n. Holotype, male, ICHUM 5034, Mx.

**Figure 13.**
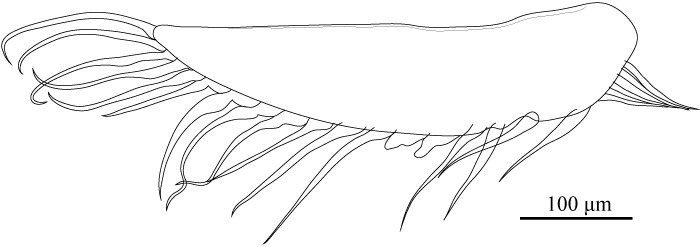
*Fabaeformiscandona kushiroensis* sp. n. Holotype, male, ICHUM 5034, vibratory plate of Mx.

**Figure 14.**
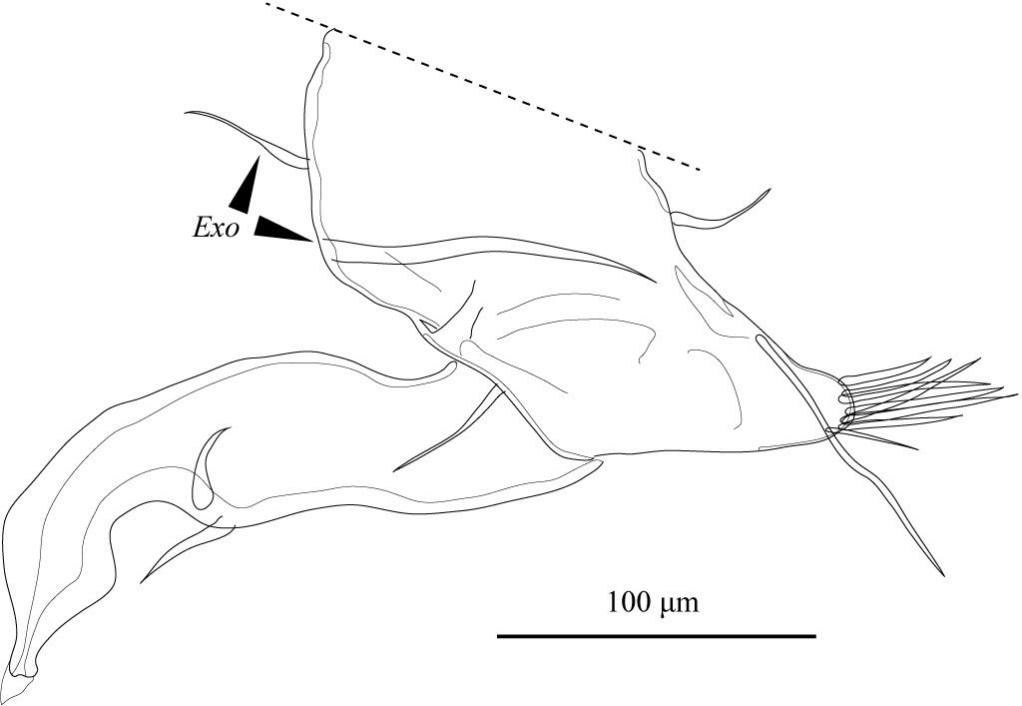
*Fabaeformiscandona kushiroensis* sp. n. Holotype, male, ICHUM 5034, Left male L5.

**Figure 15.**
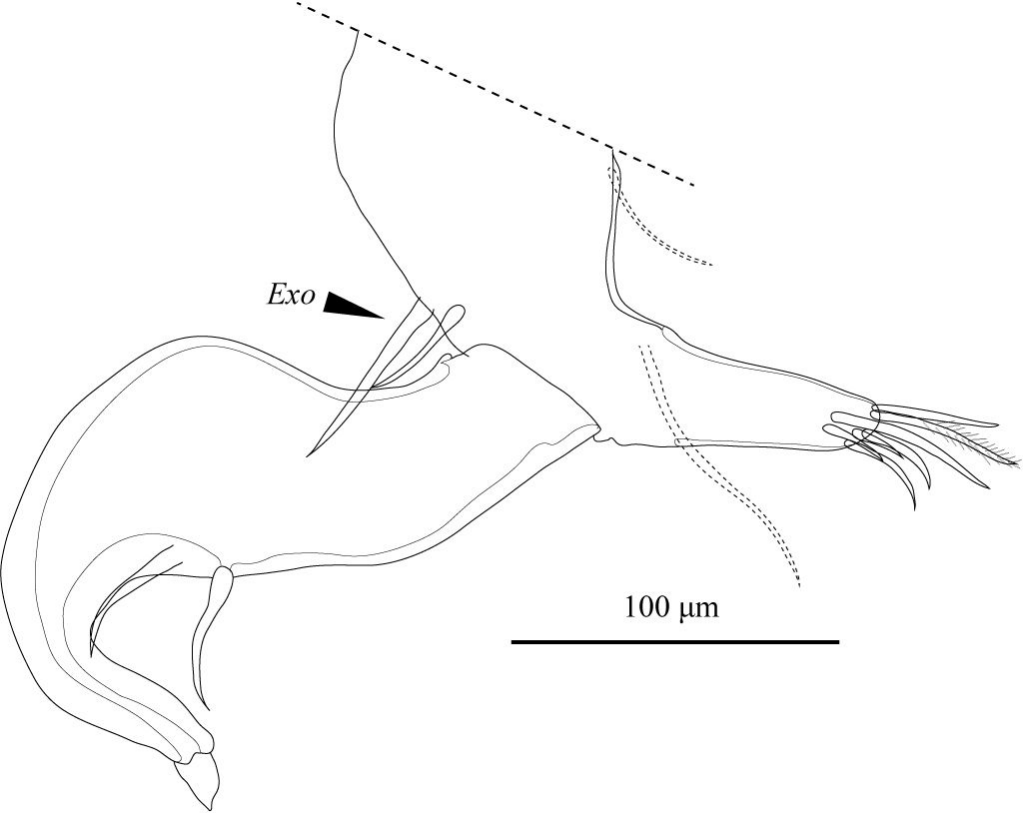
*Fabaeformiscandona kushiroensis* sp. n. Holotype, male, ICHUM 5034, right male L5.

**Figure 16.**
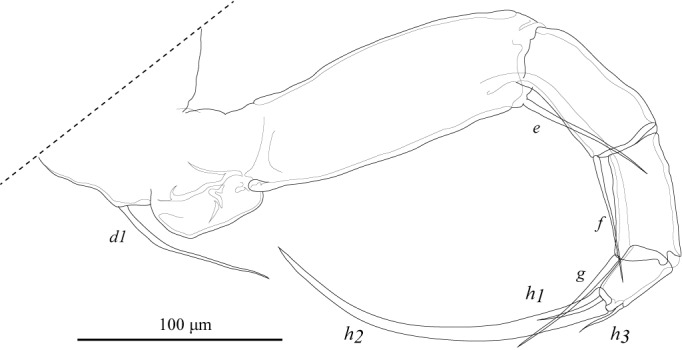
*Fabaeformiscandona kushiroensis* sp. n. Holotype, male, ICHUM 5034, L6.

**Figure 17.**
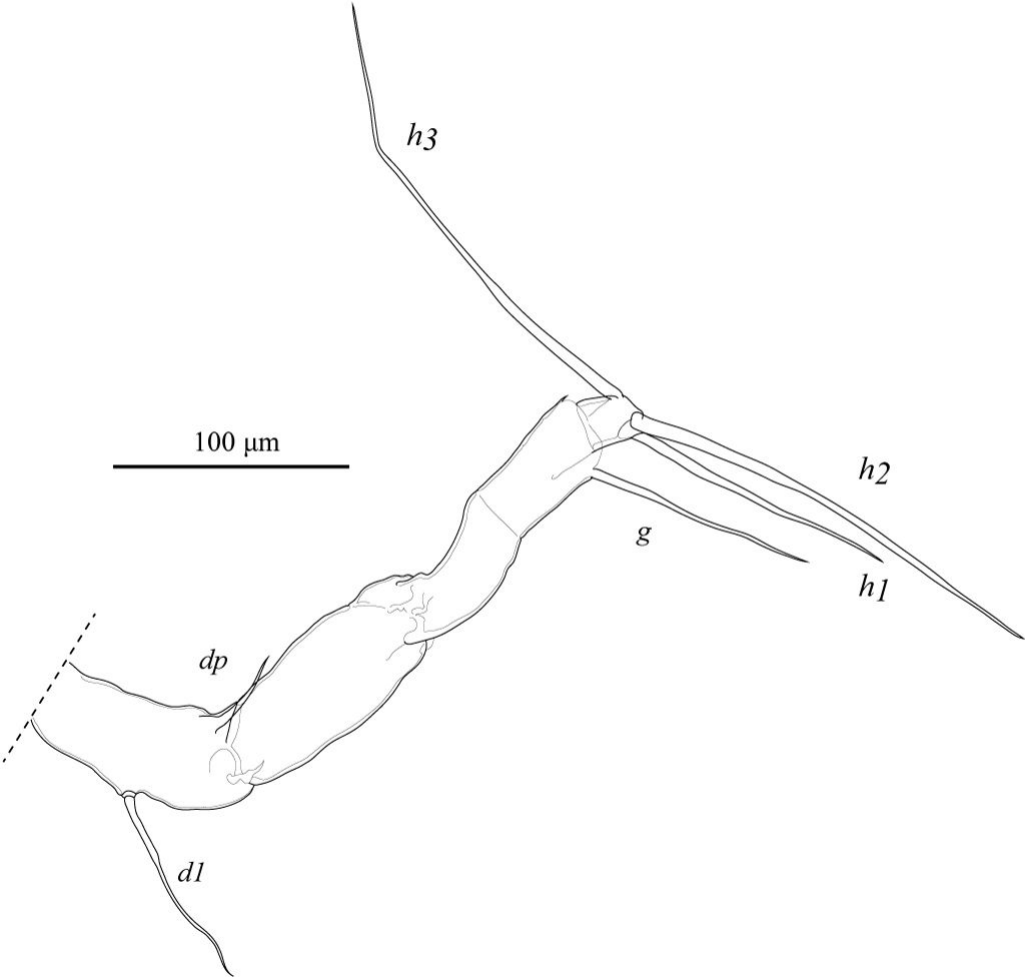
*Fabaeformiscandona kushiroensis* sp. n. Holotype, male, ICHUM 5034, L7.

**Figure 18.**
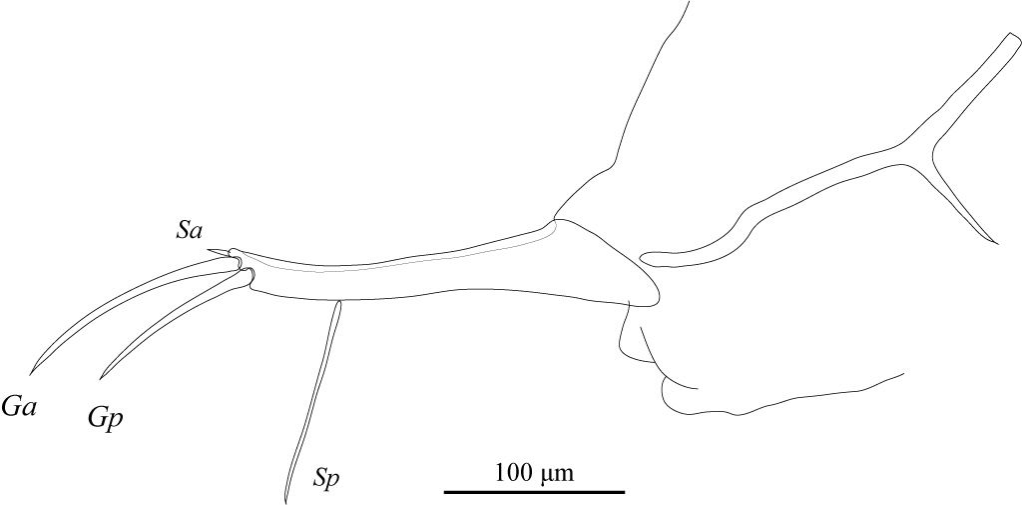
*Fabaeformiscandona kushiroensis* sp. n. Holotype, male, ICHUM 5034, Uropod.

**Figure 19.**
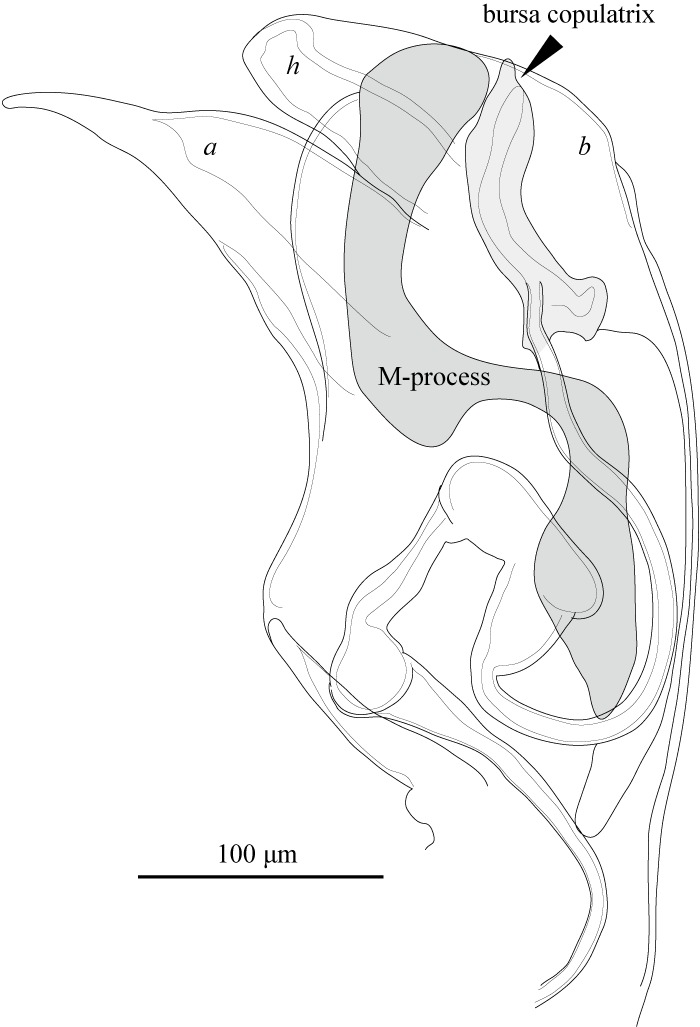
*Fabaeformiscandona kushiroensis* sp. n. Holotype, male, ICHUM 5034, Hp.

**Figure 20.**
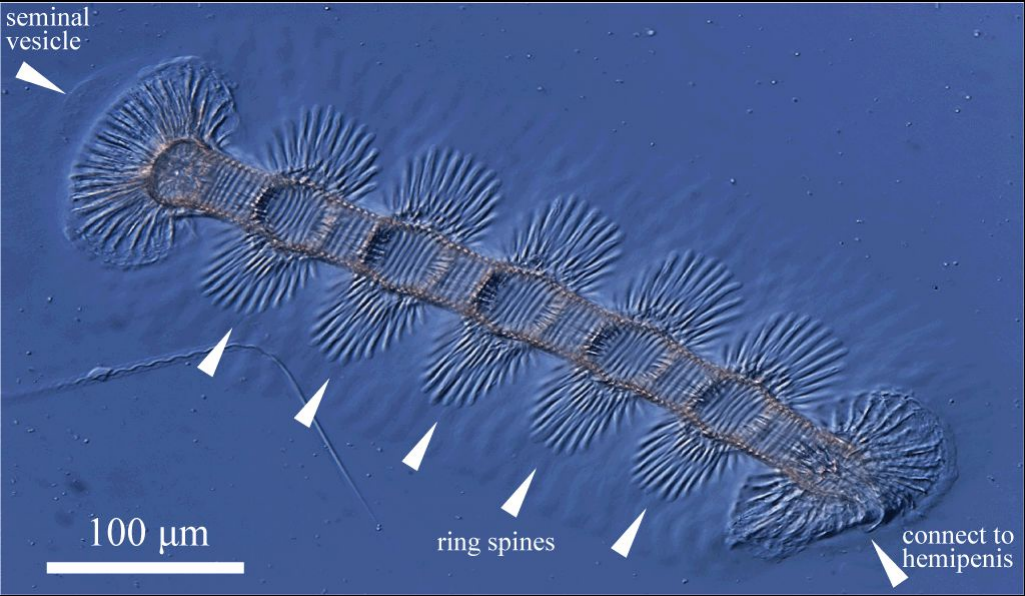
*Fabaeformiscandona kushiroensis* sp. n. Holotype, male, ICHUM 5034. Nomarski optical image of Zenker's organ.

Carapace (Figs 4, 5, 6, 8) 1.07–1.14 mm long, 0.58–0.59 mm high (n = 5; allotype 1.07 mm long, 0.58 mm high). Postero-dorsal margin of female carapace straight, with distinctive collar-like fold in right valve. Anterior margin broadly rounded, posterior margin with angular apex. Ventral margin slightly concave two-fifths of length from anterior end. Postero-dorsal flap of LV longer than that of male (Fig. 5).

**Figure 21.**
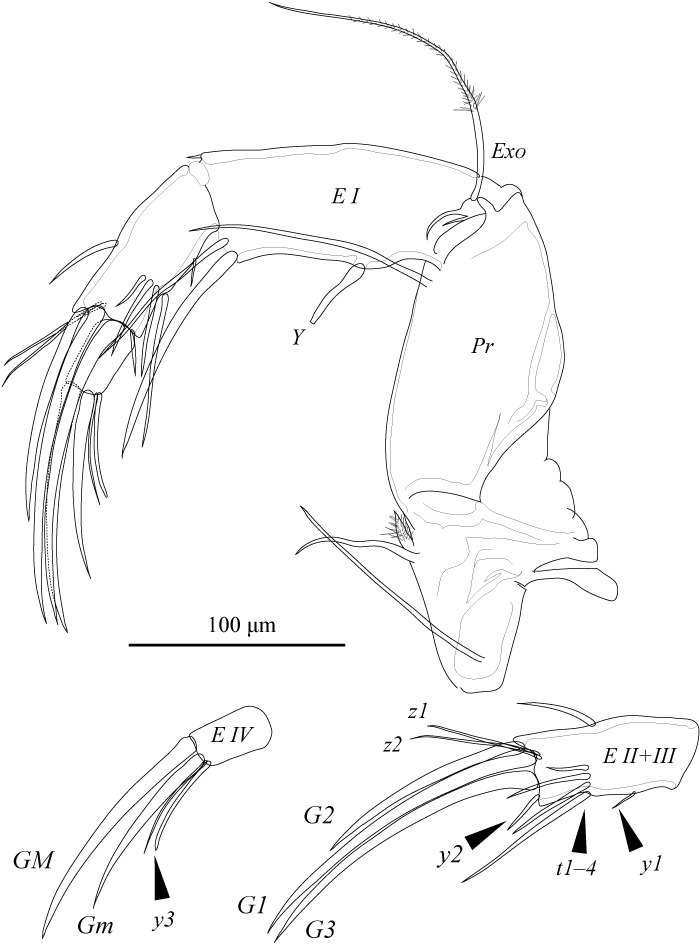
*Fabaeformiscandona kushiroensis* sp. n. Paratype, female, ICHUM 5035, Female A2 (inset details of terminal two podomeres).

A2 (Fig. 21) four-segmented. First (*Pr*) and second (*EI*) podomeres similar to those of male. Third podomere (*EII* + *EIII*) with claws *G1*, *G2*, and *z1*; short seta *G3*; longer setae *z2*, and *z3*; mid-dorsal seta; one postero-ventral aesthetasc *y2*; and four mid-ventral setae (*t1*–*4*). Fourth podomere (*EIV*) with *GM*, shorter *GM*, one apical seta, and aesthetasc *y3*.

**Figure 22.**
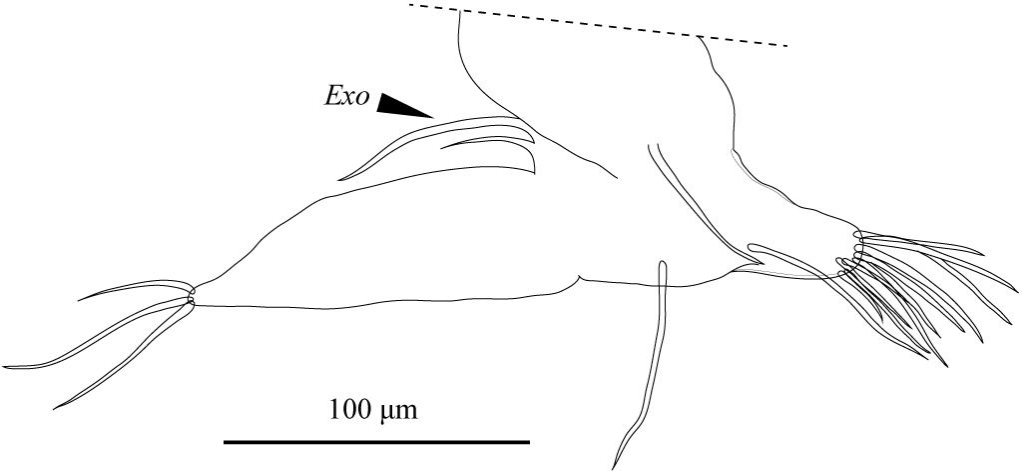
*Fabaeformiscandona kushiroensis* sp. n. Paratype, female, ICHUM 5035, L5.

Palp of L5 (Fig. 22) simple, non-segmented, with three apical setae.

**Figure 23.**
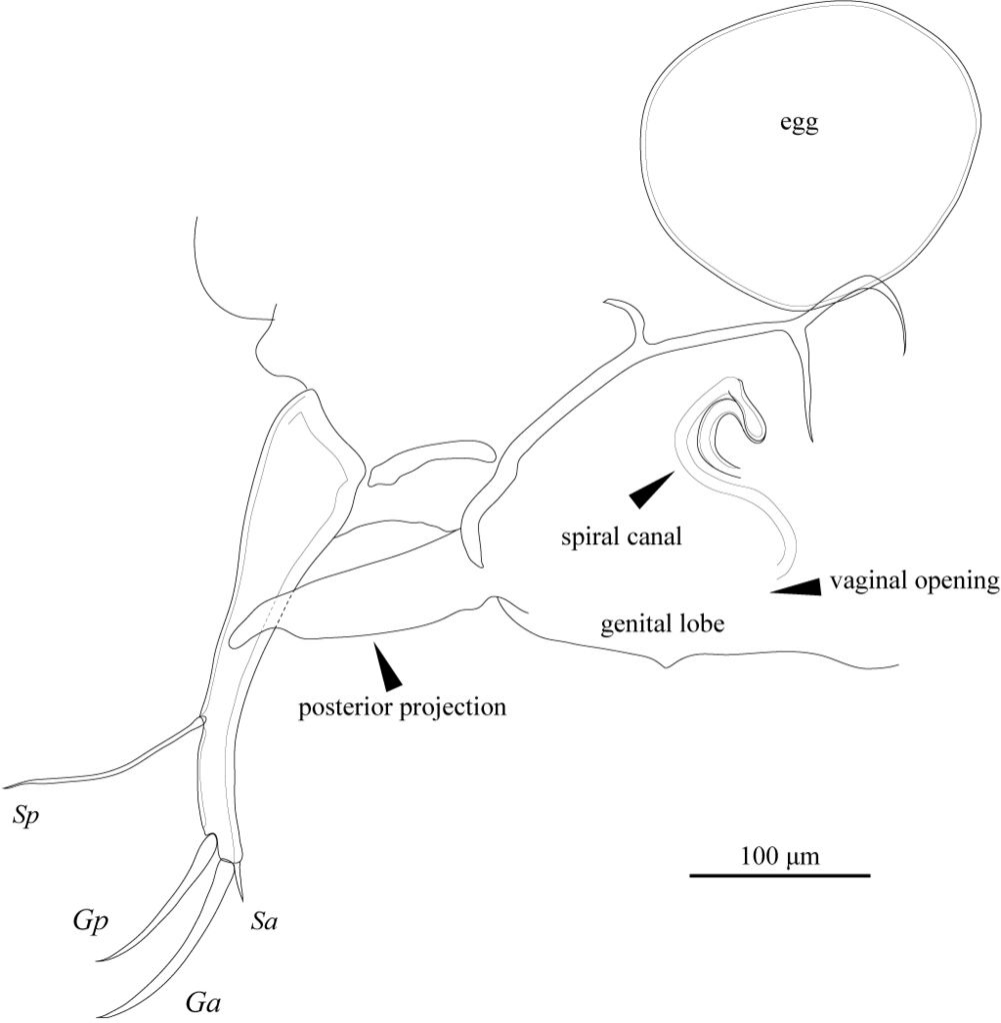
*Fabaeformiscandona kushiroensis* sp. n. Paratype, female, ICHUM 5035, Genital robe with spiral canal.

FRO (Fig. 23) with elongate, somewhat rounded posterior projection with digitiform end. Spiral canal situated anteriorly, with wall thickened near seminal receptacle. Vaginal opening inconspicuous, without rimmed chitinized ring.

In other characters, female similar to male.

#### Diagnosis

Carapace with dorsal hump one-third of length from posterior end. Male carapace has anterior and posterior margins equally rounded. Female carapace has straight postero-dorsal margin, with distinctive collar-like fold in right valve. Male hemipenis very large; medial lobe elongate, rounded distally; outer lobe elongate, with spine-like protrusion; both lobes toward dorsal side. M-process well developed, S-shaped, with rounded distal end. Projection on female reproductive organ elongate, tapering distally.

#### Etymology

The specific epithet is an adjective derived from Kushiro Marsh, type locality, in combination with the Latin suffix -*ensis*.

#### Taxon discussion

*Fabaeformiscandona kushiroensis* sp. nov. clearly belongs in the *acuminata* group (Meisch 2000), having four setae in the setal group on the second segment of the Md palp. In east Asia, the *acuminata* group contains four species, *F. danielopoli* Yin and Martens 1997, *F. nishinoae* Smith and Janz 2008, *F. akaina* Smith and Janz 2008 and *Candona quasiakaina* Karanovic and Lee 2012. Although the new species from Kushiro Marsh is generally similar to these four Asian species, these five species are clearly distinguished form each other by the following characters. 1) The posterior dorsal margin of the female RV [almost straight with a conspicuous inner fold in *F. kushiroensis*; broadly rounded in the other four species]; 2) the medial lobe (*h*) of Hp [medial lobe elongate and directed in the dorsal direction in *F. kushiroensis*; relatively small and distally digitiform in *F. danielopoli*; distally flat in *F. nishinoae*; distally square-shaped in *F. akaina*; distally large and square-shaped in *C. quasiakaina*;]; 3) M-process of Hp [distally rounded in *F. kushiroensis*; distally digitiform in the other four species]; 4) FRO [with elongate, distally digitiform process in *F. kushiroensis*; with elongate, proximally somewhat broad process in *F. danielopoli*, *F. nishinoae* and *F. akaina*; with relatively short, proximally stout process in *C. quasiakaina*]. It is generally similar to *F. hyalina* (Brady and Robertson 1870) and *F. levanderi* (Hirschmann 1912) in the shapes of the female and male valves, respectively. However, these three species differ in 1) the postero-dorsal margin of the female RV [almost straight with a conspicuous inner fold in *F. kushiroensis*; almost straight in *F. hyalina*; broadly rounded in *F. levanderi*]; 2) the distal lobes of Hp [outer (*a*) and medial (*h*) lobes elongate and directed in the dorsal direction in *F. kushiroensis*; outer lobe similar to *F. kushiroensis*, medial lobe stout and broad in *F. hyalina*; outer lobe broad and partly subdivided, medial lobe lacking in *F. levanderi*]; 3) M-process of Hp [distally rounded in *F. kushiroensis*; proximally stout and distally digitiform in *F. hyalina*; proximally stout and distally inflated in *F. levanderi*]; 4) FRO [with elongate, distally digitiform process in *F. kushiroensis*; conical in *F. hyalina*: with elongate, distally rounded process in *F. levanderi*]. Karanovic and Lee (2012) pointed out that these four species from east Asia and *F. hyalina* from Europe were closely related species in which having tapering/pointed tip on outer lobe of Hp. The new species form Kushiro Marsh also may belong to the group, because of having similar outer lobe of Hp.

#### Genome structure

*Gene contents and organization*

**Figure 24.**
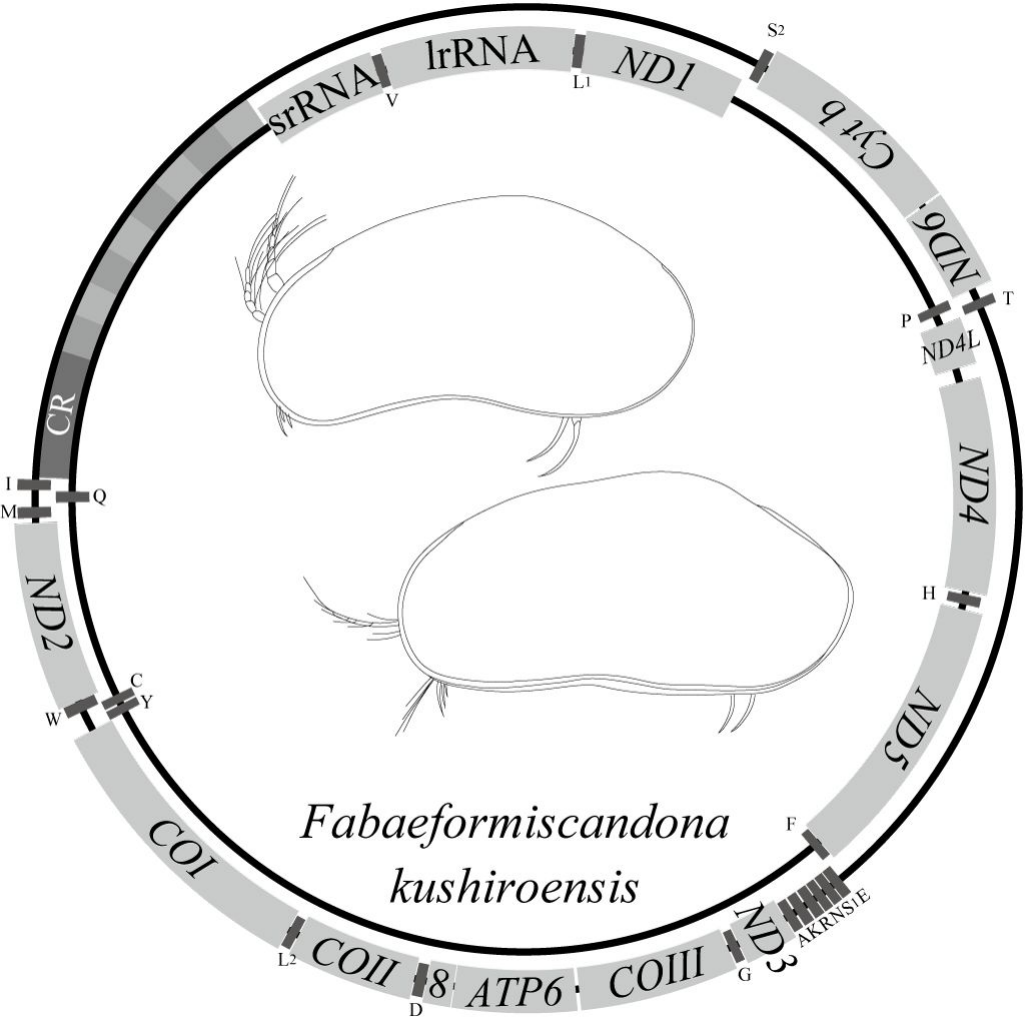
Gene map of the mitochondrial genome of *Fabaeformiscandona kushiroensis* sp. n. showing the order and the direction of 37 genes. The small bars indicate tRNA genes, labeled with single-letter amino acid designations.

**Figure 25.**
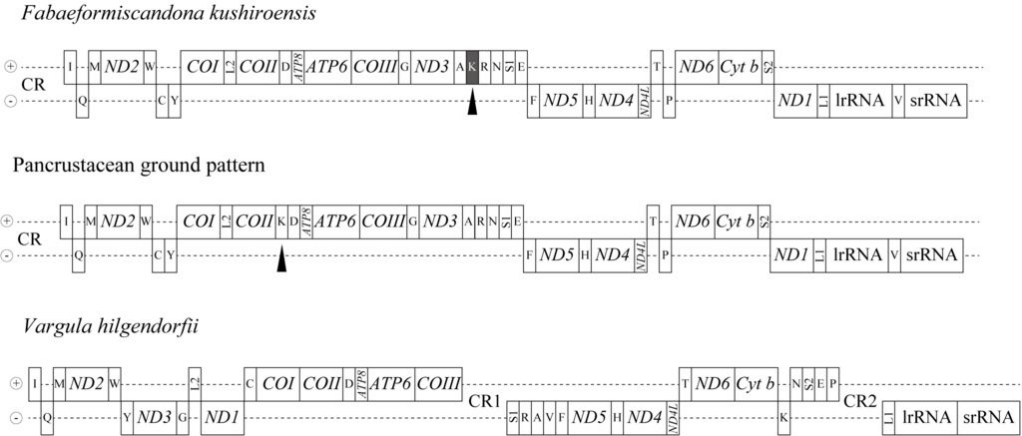
Comparison of the mitochondrial DNA gene arrangements between *Fabaeformiscandona kushiroensis* sp. n. *Vargula hilgendorfii* and hypothetical ground pattern for Crustacea. Arrowheads show position of tRNA^Lys^.

The mitochondrial genome of *F. kushiroensis* is about 17 kbp in size and includes 13 protein-coding genes, 22 tRNA genes, and two rRNA genes, as typically found in other arthropods (Fig. 24, Table 1). The order of the tRNA, rRNA, and protein-cording genes is that of the putative pancrustacean gene order (Kilpert et al. 2012), with the exception that the position of tRNA^Lys^ has been translocated to between tRNA^Ala^ and tRNA^Arg^ (Fig. 25). There is one major non-coding region that presumably contains the origin of replication and regulatory elements for transcription, as well as tandem repeat sequences. There are small gene overlaps at 12 gene borders (Table 1), with the largest (between *COIII* and tRNA^Gly^) being 50 nucleotides long. Short, non-coding sequences are also present between these genes. The largest spacer sequence (between *ND4* and *ND4L*) is 80 nucleotides long.

*Nucleotide composition*

The overall A + T content of the *F. kushiroensis* mitochondrial genome is 68.8% (A = 35.1%; T = 33.7%; G = 11.8%; C = 19.4%), which is relatively low among arthropods. Table 2 gives the A + T content for each type of gene and the CR, and Table 3 gives the nucleotide composition of each codon position for the coding genes.

*Long tandem repeat sequences in the CR*

**Figure 26.**
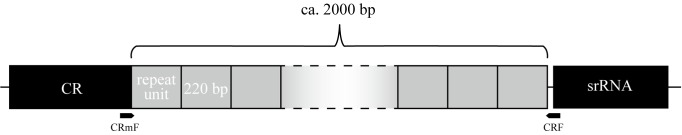
Organization of the tandem repeat region in the control region of *Fabaeformiscandona kushiroensis* sp. n.

The CR contains a long tandem repeat region composed of replicated 220-bp units, three of which were sequenced at each end of the region (Fig. 26). However the sequence of central part of the region is missing due to the technical reasons by Sanger sequencing method. The length of this region is about 2 kbp, estimated from electrophoresis.

*Transfer RNA genes*

The A + T content of the 22 tRNA genes is 72.2%, which is higher than the overall A + T composition of the mtDNA. The secondary structures of the 22 tRNA genes were determined from the MITOS Web Server to be complete cloverleaves (Suppl. material 2). The anticodon nucleotides for the corresponding tRNA genes are identical to those in *V. hilgendorfii*. The tRNA^Lys^ and tRNA^Ser(AGN)^ genes encode UUU and UCU instead of the more common CUU and GCU, respectively, typical of arthropod mtDNA. Furthermore, the tRNA^Ser(AGN)^ gene has lost the D-arm, which is also the condition in *V. hilgendorfii*. Several overlaps associated are with tRNA genes (Table 1).

*Ribosomal RNA genes*

The 12S rRNA (srRNA) and 16S rRNA (lrRNA) genes in *F. kushiroensis* are 708 and 1121 nucleotides long, respectively. Their locations and lengths were determined from an analysis of similarity to the *V. hilgendorfii* homologs and by analysis of their secondary structures reconstructed with Centroidfold (Suppl. material 3).

*Protein-coding genes*

Initiation codons in the mitochondrial protein-coding genes in *F. kushiroensis* are ATT (*ATP8*, *COI*, *COIII*, *Cyt b*, *ND3* and *ND4L*), ATA (*ND1*, *ND2*, *ND4* and *ND5*), ATC (*COII*, *ND6*), and ATG (*ATP6*) (Table 2). Twelve of the 13 protein-coding genes terminate with the stop codon TAA, whereas one (*ND1*) terminates with TAG.

*Nuclear ribosomal RNA genes*

The nuclear ribosomal RNA genes in *F. kushiroensis* are determined by a single sequence in which total 7805 nucleotides long, including internal transcribed spacer 1 and 2. Partial 18S rRNA (srRNA), complete 5.8S (lrRNA) and partial 28S rRNA (lrRNA) genes are 1792, 155 and 4007 nucleotides long, respectively. Their locations and lengths were determined from an analysis of similarity to the other sequences of Podocopid ostracods on GenBank and by analysis of their secondary structures reconstructed with Centroidfold.

## Discussion

The description of *Fabaeformiscandona kushiroensis* sp. n. brings the number of non-marine ostracods species reported from Kushiro Marsh, eastern Hokkaido, to 17 species. The specimens used in this study were collected at the end of December, from a spring area, which is a relatively temperature-stable environment.

Our samples collected at the end of December contained mature males and females, but no juveniles, which suggests that *F. kushiroensis* does not begin breeding at least before January. If *F. kushiroensis* starts breeding in early spring, its breeding strategy would be similar to that of congeners (Meisch 2000, Karanovic 2012). In Sarobetsu Marsh, northern Hokkaido, populations of *Cryptocandona* sp. in the subfamily Candoninae contain juveniles from early spring, and an increasing number of mature individuals from summer to autumn (Hiruta and Mawatari 2013).

The gene order in the mitochondrial genome of *F. kushiroensis* (subclass Podocarpa) is similar to the pancrustacean ground pattern, with just one difference. In contrast, the mitochondrial genome of *V. hilgendorfii* (subclass Myodocopa) has a quite different gene order and a duplicated CR, and its evolution from the pancrustacean ground pattern is difficult to explain through a few simple events (Ogoh and Ohmiya 2004; Fig. 25). These differences indicate a deep divergence between the subclasses Podocopa and Myodocopa, as fossil information has also suggested.

The CR in *Fabaeformiscandona kushiroensis* contains a long tandem-repeat region composed of 220-bp motif sequences. These kind of repeat region are rare not only in Crustaceans but also in other metazoans (Kilpert and Podsiadlowski 2006). While tandem repeats have been found in the CR in various organisms, they are highly variable in the length of the motifs and the number of copies. For example, the tandem repeat regions in rabbit and the fish owl contain 153 bp and 77–78 bp motifs, respectively (Casane et al. 1994, Omote et al. 2013); among arthropods, tandem-repeat sequences have previously been reported in insects and isopods (Zhang and Hewitt 1997, Kilpert and Podsiadlowski 2006).

## Supplementary Material

Supplementary material 1 Supplemental Figure 1. Data type: image
Brief description: Gene map of the mitochondrial genome of *Fabaeformiscandona kushiroensis* sp. n., showing the direction and position of PCR primers used in this study. The peripheral ring shows the two large fragments amplified with PrimeSTAR GXL and the primer sets used.
File: oo_59645.gif S.F. Hiruta

Supplementary material 2 Supplemental Figure 2. Data type: image
Brief description: Putative secondary structures for mitochondrial tRNA genes in *Fabaeformiscandona kushiroensis* sp. n., estimated with the MITOS Web server.
File: oo_59646.gif S.F. Hiruta

Supplementary material 3 Supplemental Figure 3. Data type: image
Brief description: Putative secondary structures for the mitochondrial rRNA genes in *Fabaeformiscandona kushiroensis* sp. n., estimated with Centroidfold.
File: oo_59647.gif S.F. Hiruta

Supplementary material 4 Supplemental Figure 4. Data type: images
File: oo_65602.zip S.F. Hiruta

Supplementary material 5 Supplemental Table 1. Data type: table
Brief description: List of primers used in this study.
File: oo_59656.xlsx S.F. Hiruta

XML Treatment for Fabaeformiscandona kushiroensis

## Figures and Tables

**Table 1.** Organization of the *Fabaeformiscandona kushiroensis* mitochondrial genome.

Gene	strand	Begins	Ends	Length	3' spacer	Start	Stop
Control region		1	710	710	0		
tRNA I	+	711	772	62	15		
tRNA Q	-	788	854	67	0		
tRNA M	+	855	918	64	3		
*ND2*	+	922	1923	1002	-2	ATA	TAA
tRNA W	+	1922	1985	64	-8		
tRNA C	-	1978	2037	60	6		
tRNA Y	-	2044	2105	62	-8		
*COI*	+	2098	3642	1545	-5	ATT	TAA
tRNA L2	+	3638	3700	63	0		
*COII*	+	3701	4381	681	-1	ATC	TAA
tRNA D	+	4381	4443	63	0		
*ATP8*	+	4444	4605	162	-7	ATT	TAA
*ATP6*	+	4599	5264	666	12	ATG	TAA
*COIII*	+	5277	6104	828	-50	ATT	TAA
tRNA G	+	6055	6118	64	54		
*ND3*	+	6173	6472	300	-2	ATT	TAA
tRNA A	+	6471	6531	61	0		
tRNA K	+	6532	6596	65	2		
tRNA R	+	6599	6661	63	0		
tRNA N	+	6662	6721	60	0		
tRNA S1	+	6722	6776	55	0		
tRNA E	+	6777	6840	64	2		
tRNA F	-	6843	6904	62	-26		
*ND5*	-	6879	8540	1662	51	ATA	TAA
tRNA H	-	8592	8653	62	-17		
*ND4*	-	8637	9896	1260	80	ATA	TAA
*ND4L*	-	9977	10252	276	31	ATT	TAA
tRNA T	+	10284	10347	64	0		
tRNA P	-	10348	10408	61	-43		
*ND6*	+	10366	10884	519	15	ATC	TAA
*Cyt b*	+	10900	12027	1128	1	ATT	TAA
tRNA S2	+	12029	12091	63	14		
*ND1*	-	12106	13035	930	-3	ATA	TAG
tRNA L1	-	13033	13096	64	0		
lrRNA	-	13097	14217	1121	0		
tRNA V	-	14218	14282	65	0		
srRNA	-	14283	14990	708	24		
Tandem repeated sequence		15015		ca. 2000	0		

**Table 2.** Characteristics of the mitochondrial genome of two ostracods.

Taxon	Accession number	Total length (bp)	Total	Protein-coding genes		lrRNA		srRNA		Control region	
			%A+T	AA	%A+T	bp	%A+T	bp	%A+T	bp	%A+T
*Vargula hilgendorfii*	AB114300	15923	61.6	3824	58.6	1214	70.4	714	67.9	778 + 855	67.3
*Fabaeformiscondona kushiroensis*	AP014656	16355 + α	68.8	3640	65.5	1121	77.1	708	74.0	710 + ca. 2000	78.7

**Table 3.** Nucleotide composition at each codon position in the protein-coding genes of mitochondrial genome for two ostracods.

Taxon	1st codon position				2nd codon position				3rd codon position				Overall			
	%A	%T	%G	%C	%A	%T	%G	%C	%A	%T	%G	%C	%A	%T	%G	%C
*Vargula hilgendorfii*	27.0	28.7	26.2	18.1	17.8	42.2	18.8	21.2	29.4	30.7	18.7	21.1	32.2	29.4	12.4	26.0
*Fabaeformiscondona kushiroensis*	28.1	32.0	22.8	17.1	18.3	45.0	15.3	21.2	31.6	41.0	11.7	15.5	26.0	39.5	16.6	17.9
