# Supplementary figures and images for: Description of a species of Fabaeformiscandona (Ostracoda, Crustacea) from Kushiro Marsh, Hokkaido, Japan, with the nearly complete mitochondrial genomic sequence
Source: Biodivers Data J. 2015 Dec 11;(3):e7074. doi: 10.3897/BDJ.3.e7074 (PMC4698455; doi:10.3897/BDJ.3.e7074)

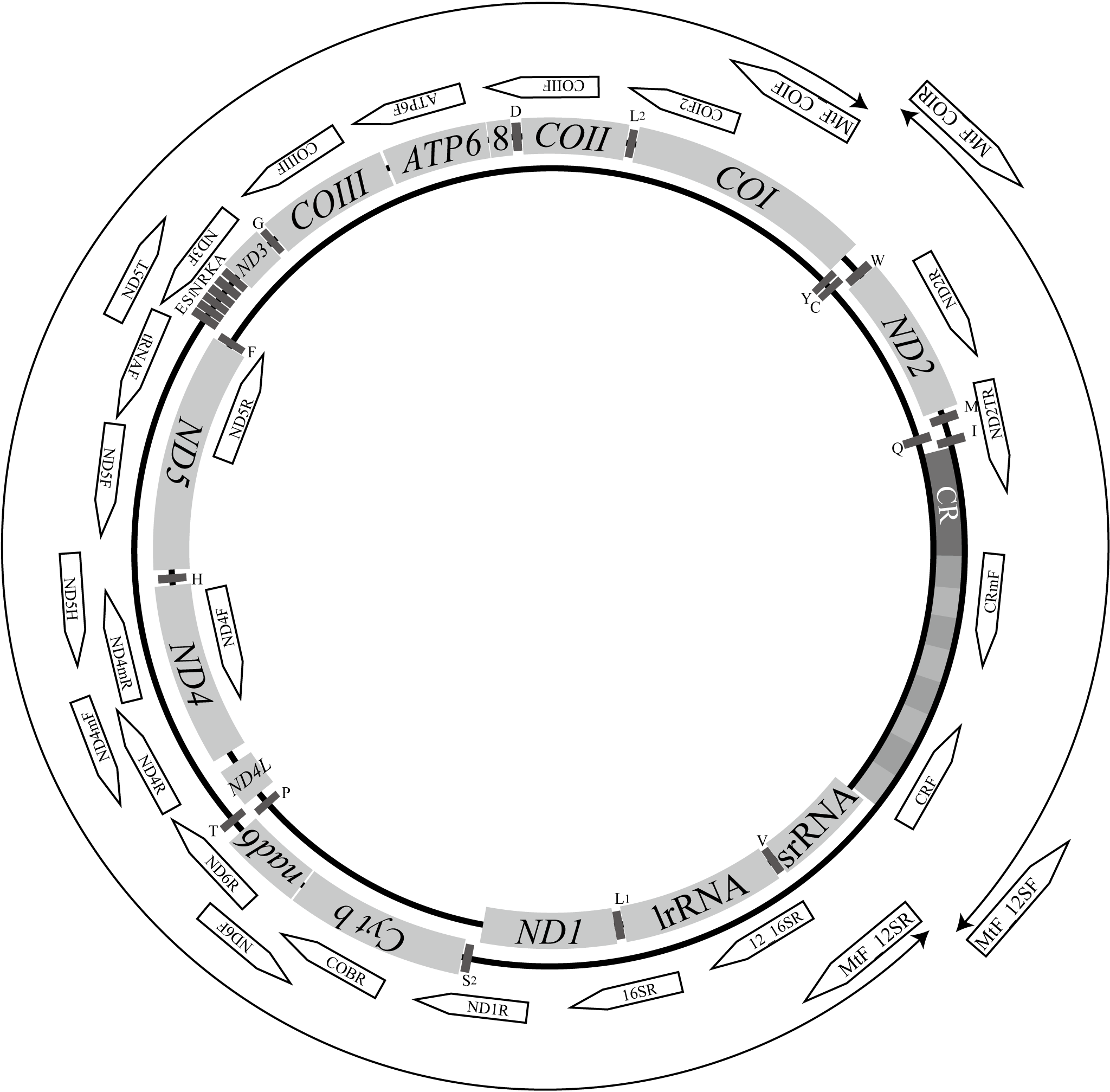

Supplement: Supplementary material 1 — Supplemental Figure 1. [file biodiversity_data_journal-3-e7074-s001.gif]

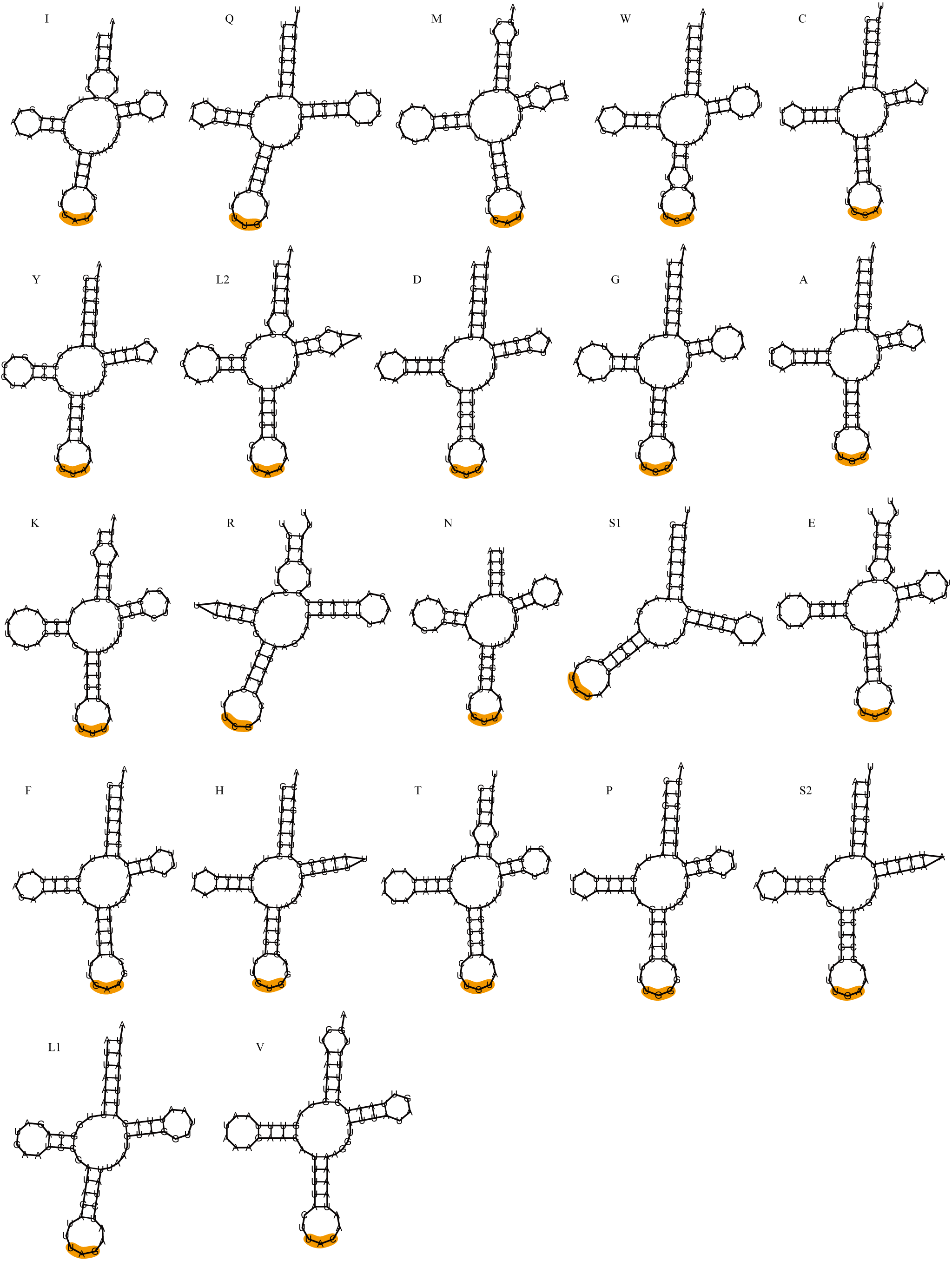

Supplement: Supplementary material 2 — Supplemental Figure 2. [file biodiversity_data_journal-3-e7074-s002.gif]

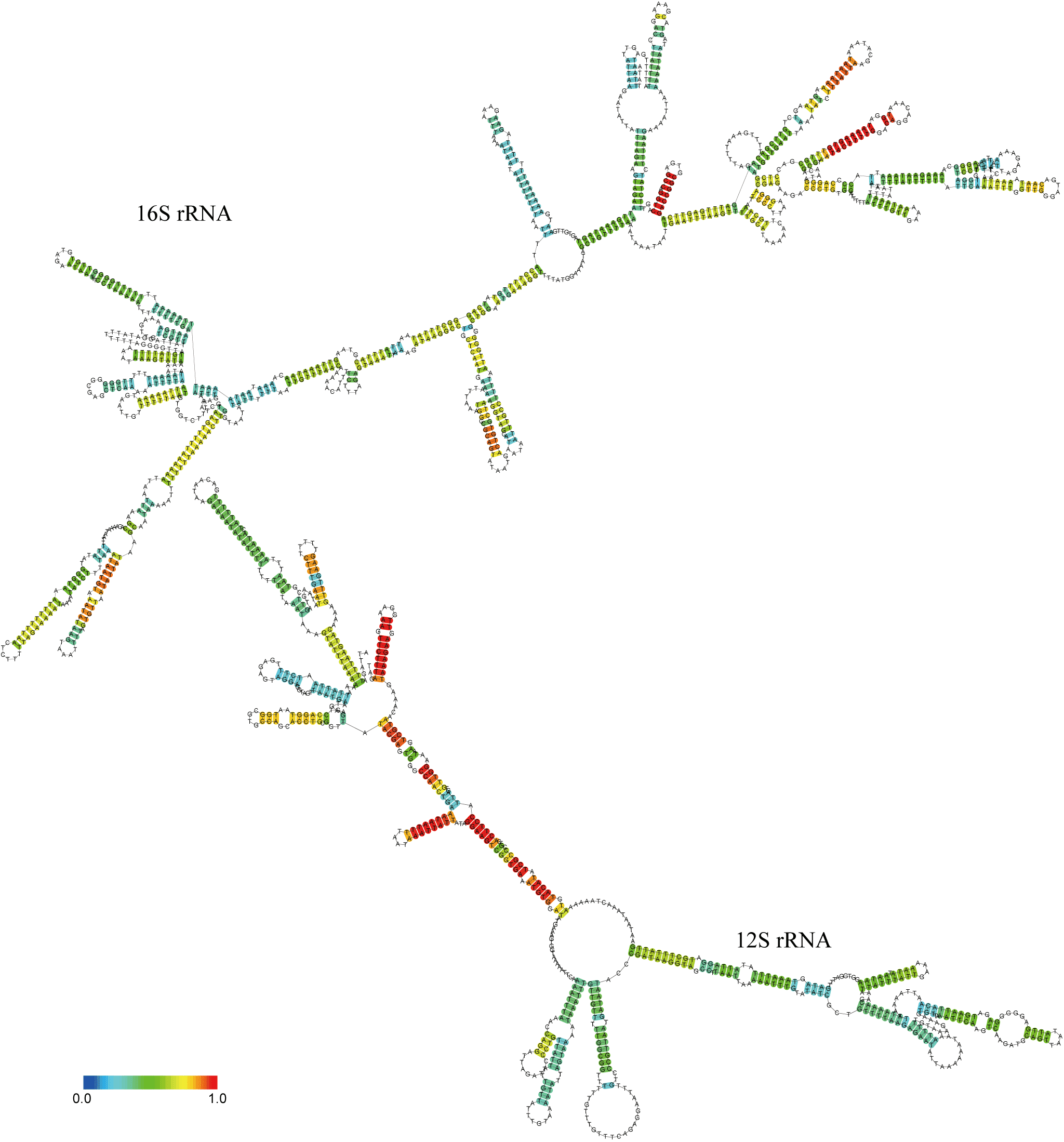

Supplement: Supplementary material 3 — Supplemental Figure 3. [file biodiversity_data_journal-3-e7074-s003.gif]

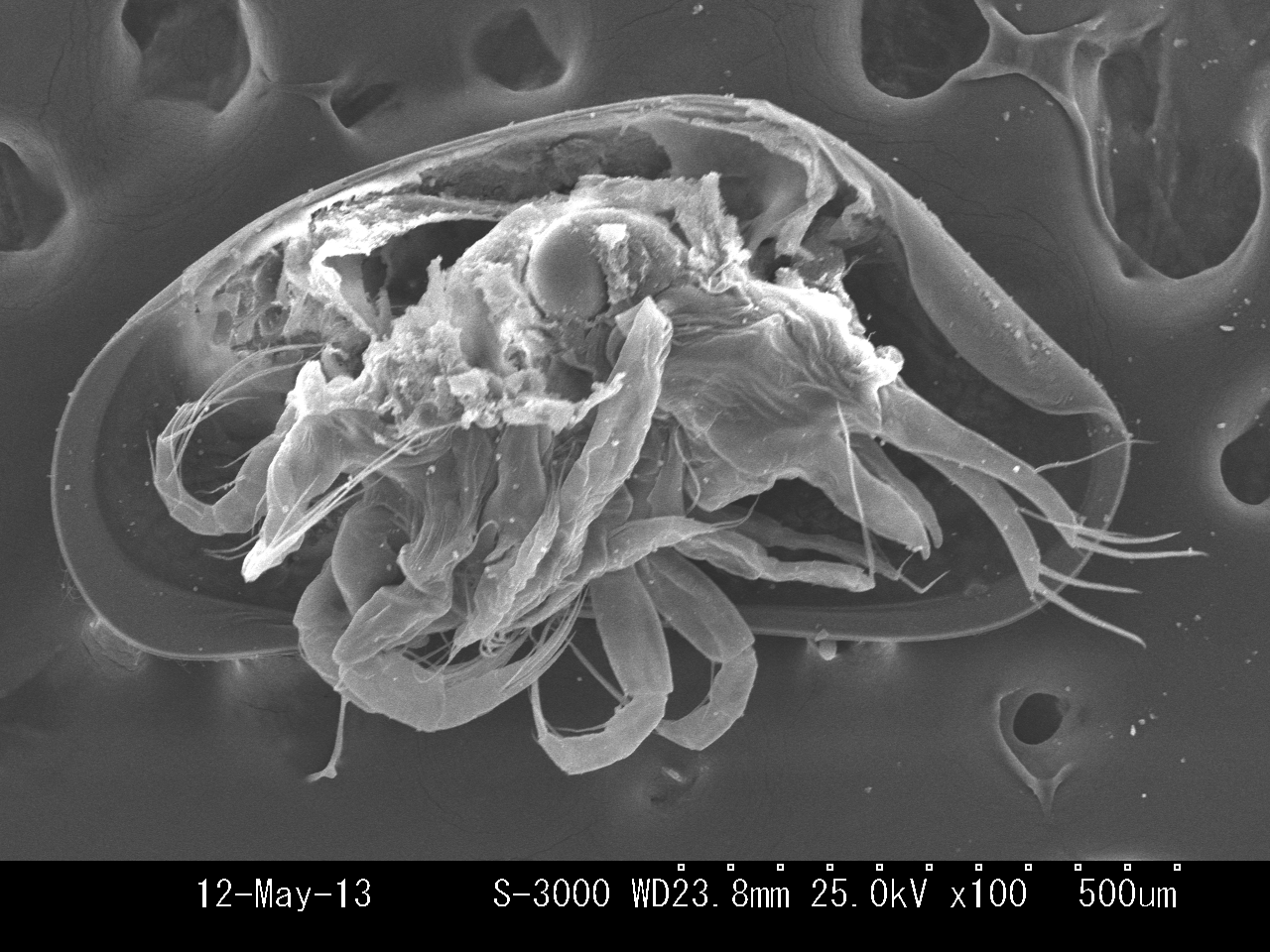

Supplement: Supplementary material 4 — Supplemental Figure 4. [file biodiversity_data_journal-3-e7074-s004.zip › Kushiro_2012Dec27_Fab_F3_Valve02.bmp]

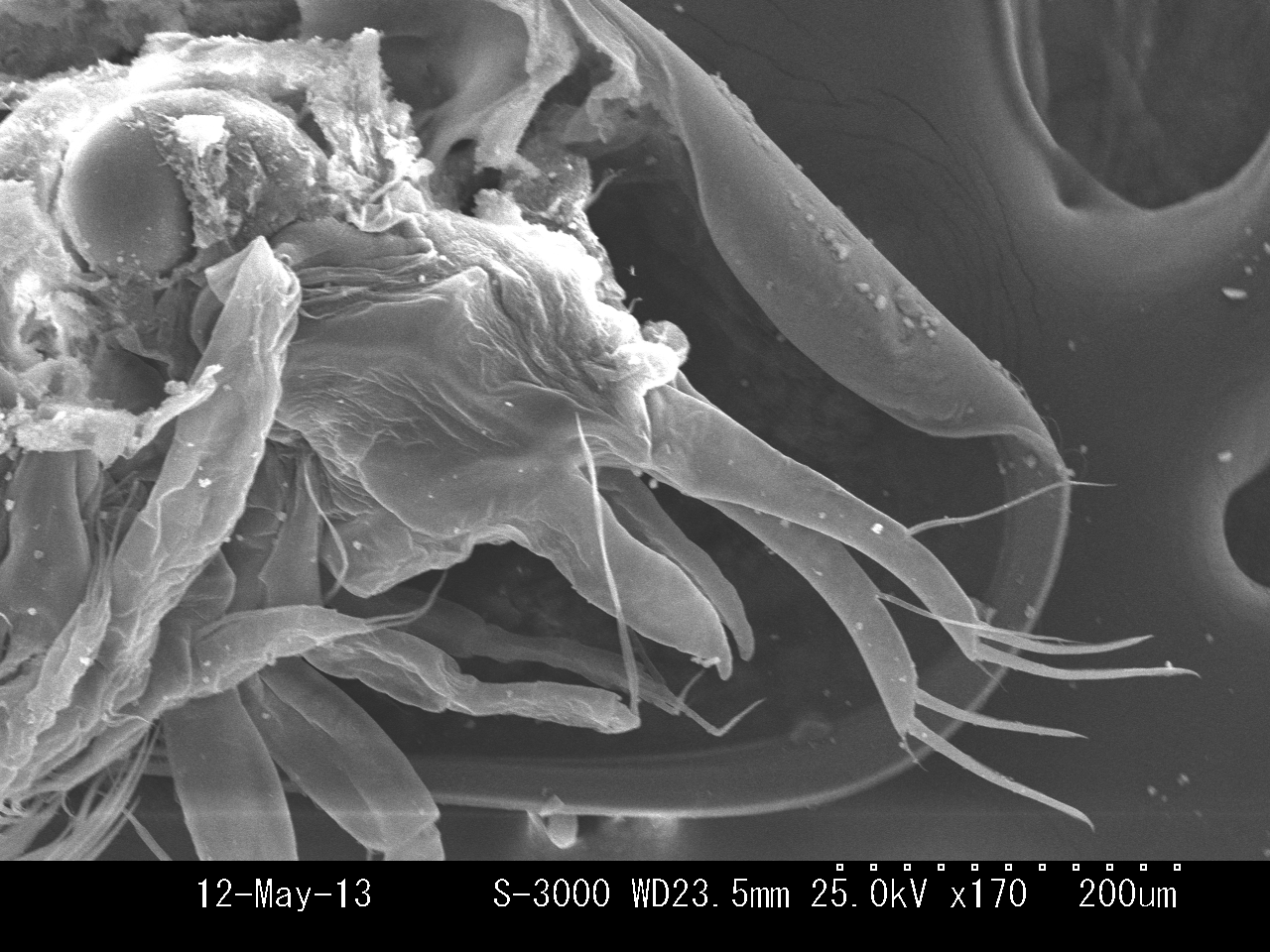

Supplement: Supplementary material 4 — Supplemental Figure 4. [file biodiversity_data_journal-3-e7074-s004.zip › Kushiro_2012Dec27_Fab_F3_Valve03.bmp]

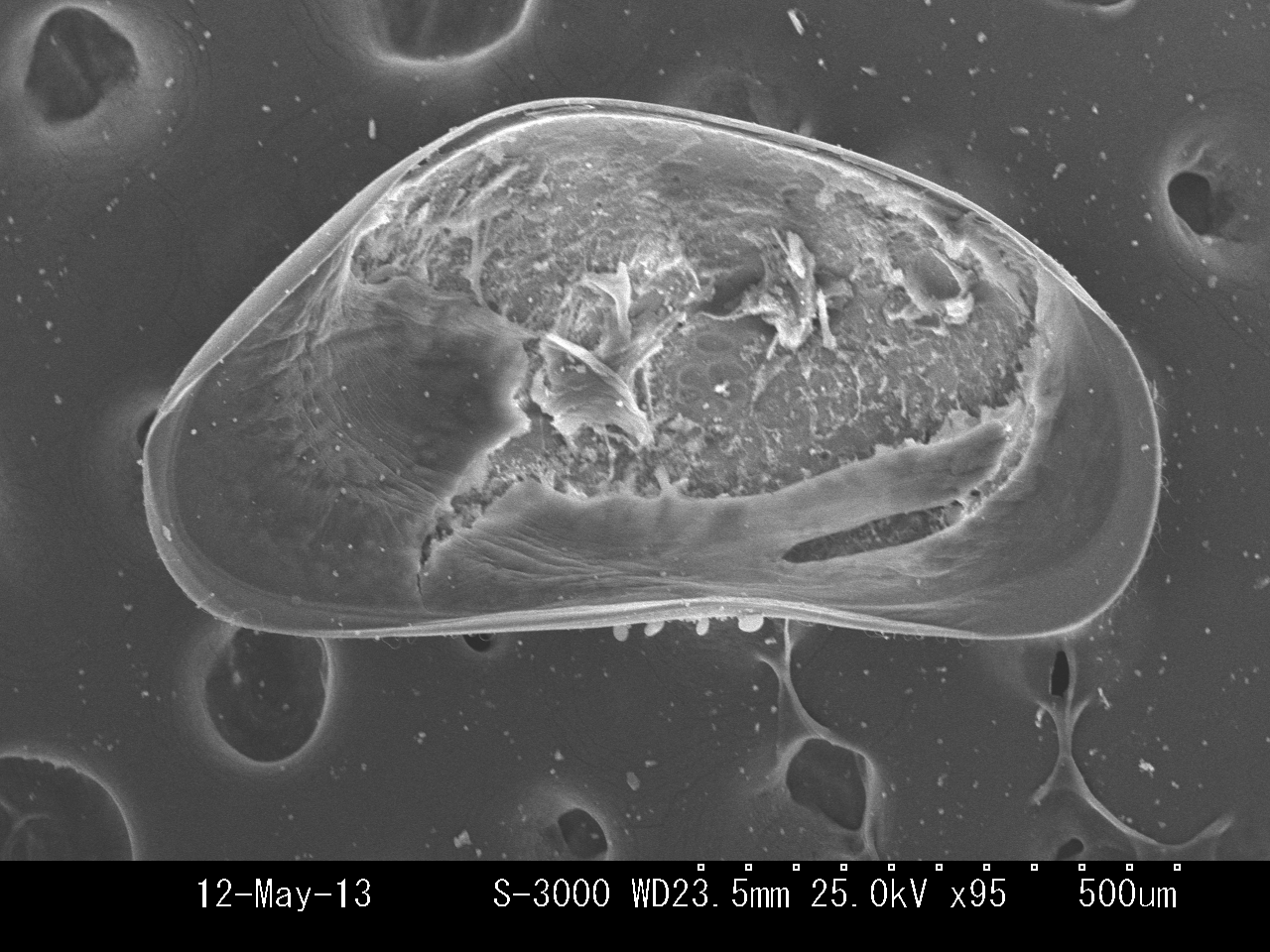

Supplement: Supplementary material 4 — Supplemental Figure 4. [file biodiversity_data_journal-3-e7074-s004.zip › Kushiro_2012Dec27_Fab_F3_Valve04.bmp]

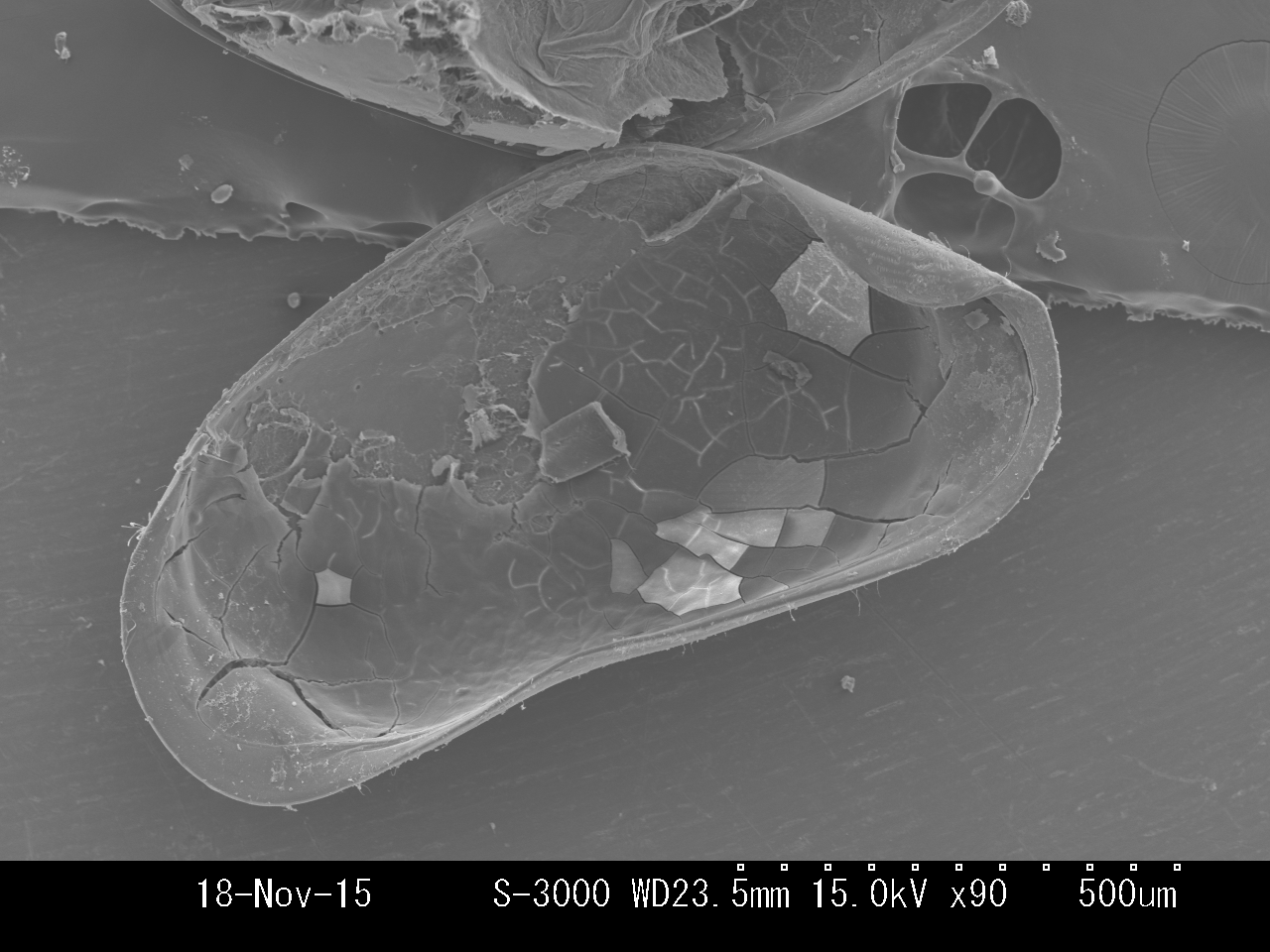

Supplement: Supplementary material 4 — Supplemental Figure 4. [file biodiversity_data_journal-3-e7074-s004.zip › Kushiro_Fab_kushiro_F04.bmp]

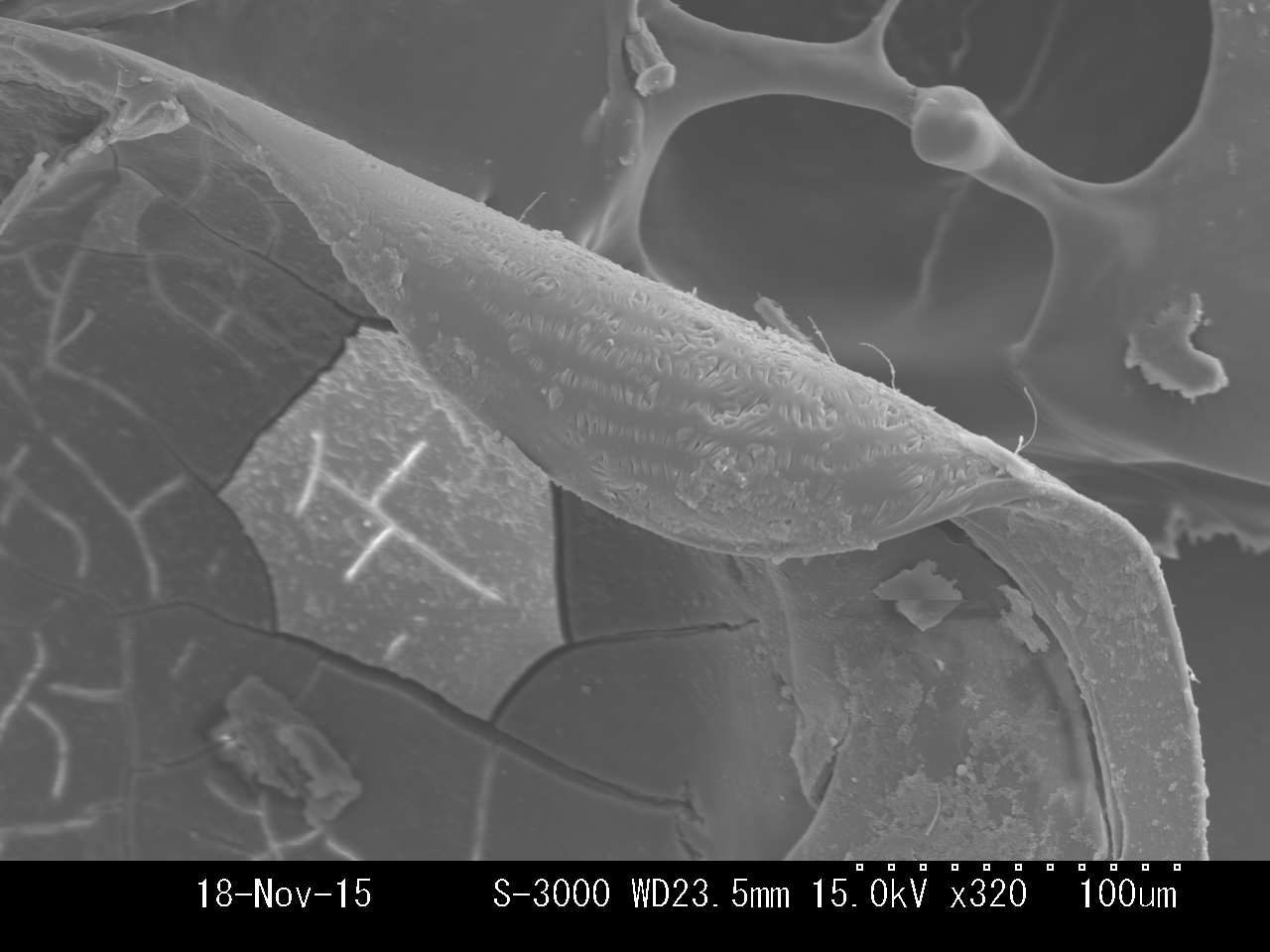

Supplement: Supplementary material 4 — Supplemental Figure 4. [file biodiversity_data_journal-3-e7074-s004.zip › Kushiro_Fab_kushiro_F05.bmp]
